# Public services and agglomeration effect under spatial structure: Threshold verification on the provincial capital cities in China

**DOI:** 10.1371/journal.pone.0321466

**Published:** 2025-05-09

**Authors:** Mei-Qi Li, Ziyan Li, Wei Zhang, Zehao Wang, Yong Zhang

**Affiliations:** 1 School of Taxation, Jilin University of Finance and Economics, Changchun, Jilin, P.R. China; 2 School of Electronic Information Engineering, Changchun University of Science and Technology, Changchun, Jilin, P.R. China.; Wenzhou University, CHINA

## Abstract

This paper explains the relationship between public services expenditure and agglomeration economy from the perspective of spatial structure, considering that the allocation of public service expenditure by local governments does not fully leverage the benefits of agglomeration effects. We constructed a general equilibrium model that examines the close relationship between public service expenditure and agglomeration effect. From a spatial structure perspective, regions are categorized into monocentric and polycentric spatial structures. We discussed the scale of urban expansion and the level of public service expenditure in secondary cities across these different types of spatial structures. It was examined 31 provincial capitals of China as research samples to validate the applicability of theoretical mechanism analysis. Six models were constructed using 2S-GMM to investigate the relationship between the public service expenditure and agglomeration effect. Meanwhile, the PTR method was employed to develop 36 models to assess the threshold effect of urban expansion scale and public service expenditure in the second city of the region, focusing on the influence mechanism of public service expenditure allocation on the agglomeration effect. Three noteworthy conclusions are as follows: (1) the scale and structure of public service expenditure has a positive influence on agglomeration effect. (2) in monocentric structure areas, we should focus on the impact of the public services expenditure structure on agglomeration effect in the changes of urban scale. (3) in polycentric structure areas, we should focus on the impact of the public services expenditure structure on agglomeration effect in the changes of scale of public services in the second central city. This research not only have enhanced the theoretical influence mechanism of public service expenditure on the agglomeration effect from the perspective of spatial structure, but also offers guidance on the allocation of public service expenditure in provincial capital cities.

## Introduction

In recent decades, especially in the context of the global economy in the 21st century, many researchers have focused on how to make more effective use of this key advantage of cities when discussing the agglomeration effect of cities [1–3]. The points have stated that the agglomeration effect of cities not only directly reflects the economic development strength of the city itself, but also reflects its economic radiation and driving ability to the peripheral areas. During the reform of China’s economic system, local governments, acting as the city agents, often utilize fiscal revenue and expenditure strategies to promote rapid economic growth, coordinating with the market economy. Particularly during times of rapid economic expansion, local governments effectively enhanced both the quality and efficiency of economic growth by increasing fiscal expenditure [4–5]. Specifically, the local governments might create agglomeration effect by increasing the scale of public services expenditure to create a good business and living environment, so as to attract more enterprises and workers to settle here, so as to obtain more agglomeration tax. [6–7]. In the meantime, the agglomeration effect of these cities could bring significant economic growth and fiscal revenue in the short time. However, under the pressure of global economic downturn and the inevitable slowdown of GDP, we have identified a phenomenon that local governments in many cities struggle with the ineffective allocation of public service expenditures, which fails to fully leverage the benefits of agglomeration. Previous studies on the allocation of fiscal expenditure focused more on the use of funds by the local governments themselves [8]. Therefore, we turn to the spatial structure closely related to the agglomeration effect [9–10]. This paper tries to incorporate spatial structure into the research, and studies how local governments allocate public services under different spatial structures to better create agglomeration effect. What’s more, this has become the real problem for this research to figure out urgently, which must be built on the basis of rigorous research hypothesis and theoretical mechanism analysis.

According to the relationship between total and combination, fiscal expenditure can be divided into the overall scale of fiscal expenditure and the structure of fiscal expenditure. The scale of fiscal expenditure refers to the total amount of fiscal expenditure arranged through the budget in a fiscal year. The scale of fiscal expenditure reflects the input capacity of governments in public services and public goods in a given period, as well as the role and influence of governments in economic and social development [11]. The structure of fiscal expenditure refers to the combination of various parts of fiscal expenditure and their quantity ratio. It reflects the allocation of fiscal funds and the priority setting in public expenditure, thus reflects the government’s policy orientation [12]. Government spending on public services, as the part of the fiscal expenditure, can be divided according to this classification.

The connotation of agglomeration economy has attracted much attention due to the vigorous development of new economic geography [13–15]. Many studies on agglomeration economy are concentrated in mechanism of agglomeration of industry [16–18] and mechanism of agglomeration of labor force [19–21]. However, from the perspective of government, this paper tries to discuss how government economic activities can be brought the agglomeration effect, as a branch of agglomeration economic theory - the mechanism of government agglomeration economy.

According to the classification of fiscal expenditure and the definition of government agglomeration effect, literature research has carried out in two aspects. (1) the influence of fiscal expenditure scale on agglomeration economy. On the one hand, the studies mainly started from regional economic growth. And a consensus conclusion has been reached: expanding the scale of fiscal expenditure can promote economic growth [22–27], especially facing the fiscal reform-oriented economic growth target [28–29], adverse economic shock [30], or stable industrialization process [31]. On the other hand, the studies focused on the spatial effect of the fiscal expenditure [32–33], which including the spatial effect of the accessibility of public service [34], the spatial effect of public services on urban agglomeration [35], the spatial effect of public sector [36–37]. (2) the influence of fiscal expenditure structure on agglomeration effect, that there are no direct papers exploring the relationship. Many studies focus on the specific fiscal expenditure which can produce agglomeration effect or promote the agglomeration economy [38–46]. Such as the agglomeration effect of high-speed rail [38], fiscal environmental protection expenditures [39–40], state-led technological innovation [41], government subsidy [42], curative health care [43], public transport services [44], housing and relocation [45–46] and so on. These studies usually use a variety of measurement methods, such as spatial Durbin model, spatial cluster analysis and so on. A few studies have focused on the agglomeration effect and public service in the city scale. Thereinto, Zhang L et al. (2023) paid attention to that public services contribute to the expansion of local cities’ scale and have a positive spatial spillover effect [47].

To sum up, public service spending can indeed have a spatial effect on the economy. The researchers of spatial economics have provided the important theoretical strength for this study. However, there are still shortcomings in the existing research: (1) there are insufficient studies on the relationship between allocation of public service expenditure and agglomeration effect; (2) the allocation of public service expenditure from the perspective of spatial structure has not been fully considered. Therefore, the research question of this article is: how local governments allocate public services under different spatial structures to better create agglomeration effect.

We have conducted the research from the theoretical analysis and empirical verification. In the theoretical analysis part, it is established the general equilibrium analysis model on the impact of public services expenditure on agglomeration effect and explored the scale and structure of public services expenditure at the same time. In the wake of dividing the region into the monocentric structure and the polycentric structure, the urban scale and competitive city (second city) has become the key factors to change the impact of the scale and structure of public services on the agglomeration effect in the core cities. In the empirical verification part, the theoretical analysis is verified by the 2S-GMM and PTR. We selected the capital cities of 27 provinces (autonomous regions) in China as research samples from 2008 to 2022. Then we used 2S-GMM to verify the effect of public service expenditure on agglomeration effect. Moreover, we used PTR to verify the threshold effect of city size and second city public service in their relationship. Ultimately, we want to solve the fiscal dilemma mentioned earlier: the optimal scale and structure of public service spending can better promote urban agglomeration. For local governments with different spatial structures in regions, facing city-scale planning or another competing city, the strategy to adjust the scale of fiscal expenditure or the structure of public services expenditure can better exert the agglomeration effect of provincial capital cities.

The research contribution includes two aspects: (1) it fills the theory of agglomeration mechanism of government economic behavior, which study the impact mechanism of allocation of public services expenditure on the agglomeration effect of cities under the spatial structure perspective; (2) through the empirical analysis of Chinese provincial capitals as research samples, this research provides guidance on the agglomeration effect of allocation of public service expenditure in provincial capital cities (core cities), based on the perspective of spatial structure. The graphical abstract is shown in S1 Fig (S1 Appendix).

The paper structure is as follows. After introduction, we have built the theoretical analysis and proposed research hypothesis in the part of Theoretical Mechanism Analysis. The part of Data and Methodology have presented the data and research methods. The samples of Chinese provincial capital cities are used for verification in the part of Empirical Results and Discussion. Finally, we get the conclusion and make further prospect in the part of Conclusions.

## Theoretical mechanism and hypothesis

**Fig 1:**
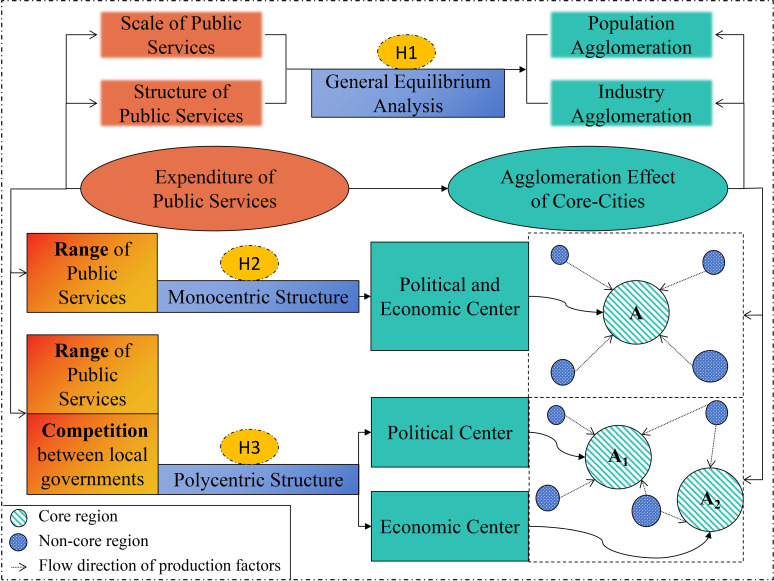
Theoretical mechanism and hypothesises of the impact of public services expenditure on agglomeration effect.

The theoretical research on public service spending and agglomeration effects begins with a general equilibrium analysis. It then explores contributions in different regional structures, specifically monocentric and polycentric regions. The theoretical mechanism of the study is shown in Fig 1.

### General equilibrium analysis

**Fig 2:**
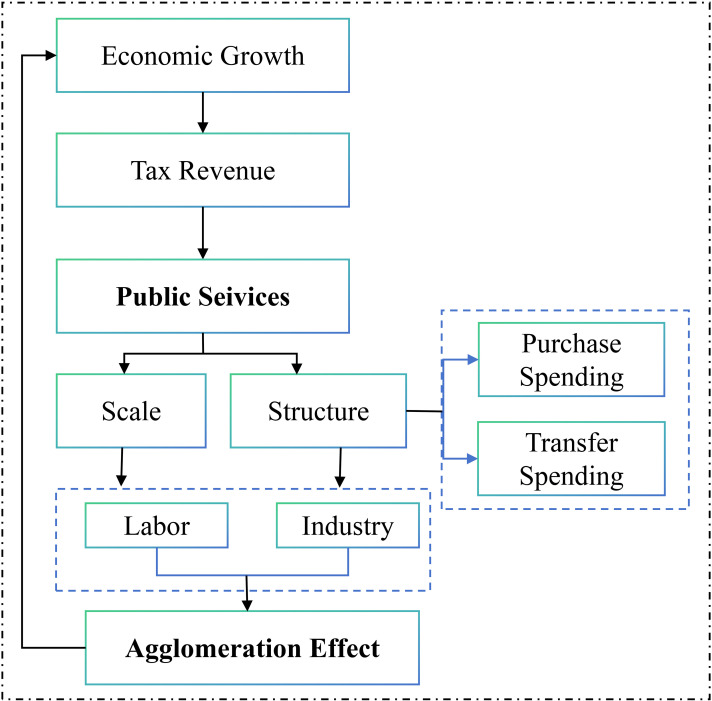
The transmission mechanism of the public services and the agglomeration effect.

The classical location theory has explored agglomeration economy earlier (Von Thunen JH, 1826; Weber A, 1929; Christaller W, 1933; Losch A, 1940; Isard W. 1956; Henderson JV, 1988) [48–53]. However, the profound insights of the agglomeration economy have become a black box for economic research because it cannot deal with the modeling. The theory of external economy (Marshall A, 1961) has explained the agglomeration economy through sharing of intermediate inputs products and labor forces and spilling of knowledge [54]. The new economic geography has endogenized the agglomeration economy and integrated it into the analytical framework of economics [13–14]. Based on this, the dynamical mechanism of agglomeration economy is the interaction between centripetal forces and centrifugal forces that determines the agglomeration level of each area ultimately [55]. To better understand the forces at play, we present the centripetal and centrifugal forces in Table 1. The forces can be considered from industry, labor force and local government. We set the local government as the protagonist, and discusses whether the government can continue to create agglomeration effect by providing public services (centripetal force), thus affecting the economic activist (labor and industry) in the agglomeration economy. Meanwhile, we need to consider negative externalities (centrifugal forces) caused by agglomeration effects that can be addressed by spending on public services. Meanwhile, the natural location and political location of a city are included in the analysis frame to discuss how different location advantages can create agglomeration effect through public service expenditure. The transmission mechanism of the public services and the agglomeration effect is shown Fig 2.

**Table 1:** The centripetal force and centrifugal force of economic subjects.

Economic Entity	Centripetal force	Centrifugal force
**Enterprise/Industry**	Higher demand from upstream and downstream firms	Competition between firms
	Easy access to cheaper intermediate input products	Intense competition in the labor market
	Higher demand from the consumer market	Higher land costs
**Labor**	High demand for labor forces	Fierce competition for positions
	Easy access to cheap consumer goods	
**Local Government**	Investment in public services	The cost of crowd including pollution, housing and high prices of non-tradable goods
	Natural and Political location advantage	
	Knowledge spillover	Tax gap

Note: The reason for knowledge spillover in local government is that the universities that produce knowledge are usually provided by the government under our assumptions.

One of the governing goals of the local governments is the economic growth of the region, which is also one of the important indicators in the promotion championship [56–57]. In the hands of local governments, tax reduction and public spending increase are the common tools used to stimulate economic growth [58]. This research focuses on public services spending. We divide public services spending into two categories. One is how much fiscal funds the government can take out to public services spending (Scale). The other is the percentage of the public service expenditure. We divide the public service expenditure into two part-- purchase expenditure and transfer expenditure. The percentage of purchase expenditure and transfer expenditure are the allocation of fiscal funds by the government (Structure).

In the regional economic growth, we have observed that governments can attract more enterprises and labor force by investing a large number and a wide variety of public products into the local area, thus producing more agglomeration effect, promoting economic growth, further increasing tax revenue and fiscal funds. This kind of behavior can also attract labor force to enter and improve labor skills, so as to fully realize the agglomeration effect [59]. In previous empirical studies, urban size distribution in a region is closely related to population size. [60]. Based on the above relationship, local government’s investment in public services would establish a close relationship with city size through population size.

There is a strong relationship between the proportion of various services and the needs of local residents and enterprises [61–63]. Aiming at pursuing economic growth and promotion, local governments adjust the structure of public service expenditure to meet local economic activities [64–65]. On the one hand, as the agglomeration economy expands, local governments have to face the cost of crowded and need to cost more public fiscal funds to manage the cost of congestion on account of attracting too many firms and too many people. For example, expanding the regional boundaries of the city to accommodate more industries and people, and adding urban expressways to combat traffic jams [66]. This condition would force governments to restructure public services. On the other hand, if local governments face the competition from neighboring cities or cities with similar political functions, they would adjust the allocation strategy of fiscal funds to counter the competition due to the limitation of fiscal funds [67]. Based on the above discussion, we hypothesize that there are two factors that the public goods affect the agglomeration effect. One is the scale of public goods investment that can match the scale of the city. The other is the structure of public goods investment that can match the local economic activities. The Hypothesis 1 of the general equilibrium analysis is as follows:

#### Hypothesis 1.

The contribution of the expenditure of public services to the agglomeration effect of cities is closely related to the scale and structure of expenditure.

### Monocentric structure region

In the new economic geography, the typical spatial form between two regions is the regional model of Center-periphery model. In this form, the model of the initial urban hierarchy system is monocentric structure [68]. All economic activities are closely centered around the single core city. By a province, a single core city in the region is usually both the political center (the capital city) and the economic center (the level of GDP is the highest in the region). What about the contribution of public goods input to agglomeration effect of the core city?

Generally, the scale and structure of the public services in the single core city are the best in the province, so that the agglomeration effect of the city inevitably plays a crucial part [69]. In the short term, the agglomeration effect can be reified as the core city attracts labor and industry within the province. However, in the long term, the core city may have negative externalities because of congestion and pollution. Labor and industry have to experience poorer living environment, higher cost of living and production. At the moment, the local governments of core cities might have two strategies. The one is governing negative externalities which is needed more government spending. The other one is expanding the land area of the urban area to ease traffic congestion and so on, which is needed to be added more public services. The impetus for local governments to choose the latter strategy is the transfer of land-use rights (in exchange for more fiscal funds) and the creation of employment and GDP (from the expenditure of the new public services) [70]. From the past experience of urbanization in China, the speed of land urbanization was much faster than the speed of population urbanization [71]. As a result, it can be found that land prices are cheaper in places farther away from cities, but the land revenue and fiscal supplement from the agglomeration effect cannot match the increase in new public service expenditures, and the long-term fiscal balance cannot be sustained [72]. Based on the above discussion, we hypothesize the following that the effect of public service expenditure on agglomeration effect needs to bring into the degree of urban expansion. Hypothesis 2 of monocentric regional structure is as follows:

#### Hypothesis 2.

In the monocentric regional structure, the strongest agglomeration effect needs a matching interval between the public services expenditure and urban expansion of the core city.

### Polycentric structure region

In the new economic geography, the emergence of new cities is the breakdown of the equilibrium of the original urban system, thus forming a new urban system [73]. At present, there is an explanation in evolution theory for the polycentric structure region, that the subcentral city, where the population and industry begin to flow because of the negative external effect overload of agglomeration brought by the monocentric structure, could evolve as the other center in the region [74–75]. This is the process and result of the operation of the free market mechanism. But in fact, there is another explanation that some cities where has some advantages such as coastal location or certain policy preferences have become the economic centers of the region [76]. After the political center and the economic center are separated in a province, what is the impact mechanism of the public services expenditure on the agglomeration effect of the provincial capital city?

Local governments in both core cities would compete for more agglomeration economies. It would inevitably make them provide more larger scale and variety of public goods to improve urban competitiveness such as the similar levels of medical services, the same number of universities, and complete infrastructure construction in both political center and the economic center cities [77]. Moreover, in order to strive for more labor inflow and industry entry, the local governments of the core city could be not scruple to sacrifice local tax revenues and invest more public spending. In the two-center structure, if the vicious competition for the agglomeration economy between the political center and the economic center cannot be solved, the contribution of the public services expenditure of the core city on the agglomeration effect would be decomposed by the other center city. Based on the above discussion, we hypothesize that the contribution of fiscal expenditure to the local agglomeration economy is not efficient all the time in the polycentric structure. In particular, the provincial administrative divisions in China have included not only provinces and autonomous regions, but also municipalities directly under the central government, where have important impacts on the economy of the surrounding regions. Therefore, the polycentric structure regions that we would define are including the provincial capital adjacent to the municipality in the following empirical study. Hypothesis 3 of polycentric structure region is as follows:

#### Hypothesis 3.

In the polycentric structure region, the contribution of public services expenditure of core city to the agglomeration effect is closely related to the public services expenditure of the other core city in a province.

## Data and methodology

### Data

There are four types of provinces in China, including municipalities directly under the central government, provinces, autonomous regions and special administrative zones. This paper has focused on provinces and autonomous regions with a large number of cities and land scale and taken into account the availability of data in the meantime, which are 22 provinces and 5 autonomous regions. The selection of research sample is shown in Table 2. It is worth mentioning that we have specifically marked the structural characteristics of provinces or autonomous regions whether it is monocentric structure (M) or polycentric structure (P) in the Table 2. Meanwhile, in order to make empirical analysis accurately, we have listed the cities which are the second economic level only to the provincial capital city in monocentric structure regions, and the economic centers or the adjacent municipalities directly under the central government in polycentric structure regions. The division method is shown in S2 Fig (S1 Appendix).

**Table 2:** The selection of research sample.

Provinces and Autonomous Regions	Provincial Capital	M/P	Second City	Number of Prefecture-level Cities
**Anhui**	*Hefei*	M	Wuhu	16
**Fujian**	*Fuzhou*	P	Quanzhou	9
**Guangdong**	*Guangzhou*	P	Shenzhen	21
**Gansu**	*Lanzhou*	M	Qingyang or Jiuquan	14
**Guangxi**	*Nanning*	M	Changzhi	14
**Guizhou**	*Guiyang*	M	Zunyi	9
**Hainan**	*Haikou*	M	Sanya	4
**Hebei**	*Shijiazhuang*	P	Beijing and Tianjin	11
**Henan**	*Zhengzhou*	M	Luoyang	17
**Heilongjiang**	*Harbin*	M	Daqing	13
**Hubei**	*Wuhan*	M	Yichang or Xiangyang	13
**Hunan**	*Changsha*	M	Yueyang	14
**Inner Mongolia**	*Hohhot*	P	Ordos	12
**Jilin**	*Changchun*	M	Jilin	9
**Jiangsu**	*Nanjing*	P	Shanghai	13
**Jiangxi**	*Nanchang*	M	Ganzhou	11
**Liaoning**	*Shenyang*	P	Dalian	14
**Ningxia**	*Yinchuan*	M	Shizuishan or Wuzhong	5
**Qinghai**	*Xining*	M	Haidong	8
**Shandong**	*Jinan*	P	Qingdao	16
**Shannxi**	*Xian*	M	Yulin	10
**Shanxi**	*Taiyuan*	M	Changzhi	11
**Sichuan**	*Chengdu*	P	Chongqing	21
**Xinjiang**	*Urumchi*	M	Yili	14
**Xizang**	*Lhasa*	M	Changdu	7
**Yunnan**	*Kunming*	M	Qujing	16
**Zhejiang**	*Hangzhou*	P	Shanghai	11

Note: In this table, the third column is marked about the monocentric structure (M) or the polycentric structure (P) that there is one city which has a higher level of GDP than the provincial capital city of the region. The fourth column is the other city which is represented two meanings: (1) provincial capitals adjacent to municipalities (Beijing, Chongqing, Shanghai and Tianjin) are marked; (2) the second city in monocentric structure regions are listed as two because the second economic center of a province replaces each other in some years. The fifth column has shown the number of cities in a province at the same administrative level as the capital city.

The original data derived from the statistical yearbooks of 22 provinces, 5 autonomous regions, 4 municipalities directly under the central government, and 27 provincial capital cities from 2009 to 2023, and some missing data can be obtained from the statistical bulletin of national economic and social development of cities from 2008 to 2022.

### Definition and calculation of variables

The method of calculation for all variables are shown in Table 3. From the view of the overall agglomeration effect and the categorize types of agglomeration effect, there are three explained variables, which are Agglomeration Effect (*AE*), Industrial Agglomeration Effect (*IAE*) and Population Aggregation Effect (*PAE*). The explaining variables are included the scale of fiscal expenditure (*FEscale*) and the structure of fiscal expenditure (*FEstructure*) on public services. The relationship between AE and FE is shown in S3 Fig (S1 Appendix).

**Table 3:** The calculation methods for the variables.

Variables Type	Variable	The Method of Calculation
**Explained Variable**	*AE*	$\frac{{GDP}_{i,t}}{{GDP}_{provience,t}}$
	*IAE*	$\frac{{The\;Sum\;of\;the\;Secondary\;and\;Tertiary\;Industries}_{i,t}}{{The\;Sum\;of\;the\;Secondary\;and\;Tertiary\;Industries}_{provience,t}}$
	*PAE*	$\frac{{Population}_{i,t}}{{Population}_{provience,t}}$
**Explaining Variable**	*FEscale*	$\frac{{Fisacl\;Expenditure}_{i,t}}{{Fisacl\;Expenditure}_{provience,t}}$
	*FEstructure*	*PS* =Purchase Spending(a)+Purchase Spending(b)
		${FE}_{structure}=\frac{{PS}_{i,t}}{{TS}_{i,t}}$
**Threshold Variable**	*Urban*	$\frac{{Unban\;Population}_{i,t}}{{Overall\;Population}_{i,t}}$
	*FEother*	$\frac{{Fisacl\;Expenditure}_{i,t}}{{GDP}_{i,t}}$
**Control Variable**	*LGS*	$\frac{{Fiscal\;Revenue}_{i,t}}{{GDP}_{i,t}}$
	*IS*	$\frac{{The\;Value-added\;of\;Secondary\;Industry}_{i,t}}{{The\;Value-added\;of\;Tertiary\;Industry}_{i,t}}$
	*Open*	$\frac{{Total\;Export-Import\;Volume}_{i,t}}{{GDP}_{i,t}}$
	*PI*	$\frac{{Population}_{i,t}}{{Area\;of\;Land}_{i,t}}$

Note: In the subsequent grouping of spatial structures, the variables are calculated in the same way as in this table. The specific types of PS and TS are placed in S12 Table (S1 Appendix).

The threshold variables that we have chosen two types are as follows: (1) the urban size (*Urban)* is the first threshold variable for the public services to affect agglomeration effect; (2) the public services expenditure of the second city (*FEother)* in a province is the second threshold variable for the public services expenditure to affect agglomeration effect of the provincial capital city.

The control variables which we have chosen are four, as follows:

(1) Local Government Scale (LGS) is represented the administrative operation capacity of local government with the tax revenues. Local governments usually play a vital role in economic development. The scale of local government, namely its fiscal capacity, administrative structure, directly affects the government’s ability to provide public services, and carry out infrastructure construction, and formulate and implement economic policies. These aspects are the key factors affecting the development of agglomeration economy.
(2) Industry Structures (*IS*) is represented the compatibility between the secondary industry and the tertiary industry of the provincial capital city. Industrial structure reflects the proportion of different industries in the economy and the relationship between them. The optimization and upgrading of industrial structure are the important driving force to promote economic development and one of the key factors affecting agglomeration economy. The demand of different industries for resources, the difference in production mode and the extension of industrial chain would affect the formation and development of agglomeration economy.
(3) Foreign Trade Level (Open) is represented the economic openness of the provincial capital city with the total export-import volume. The improvement of the level of foreign trade means a wider international market and more international resources can be utilized, which helps to promote the development of agglomeration economy.
(4) Population Intension (*PI*) is chosen to represent the number of people in per unit of land area because of the differences between the provincial capital cities. Population density is an important index to measure the density of population distribution in an area. The change of population density directly affects the supply of labor resources, market demand and consumption level, and then has an impact on agglomeration economy. S1–S11 Tables (S1 Appendix) have shown the data of all variables. The descriptive statistics of each variable is shown in Table 4.

**Table 4:** The descriptive statistics for the variables.

Spatial Structure Type	Variable	N	Mean	St	Max	Min
***General Equilibrium***	*AE*	405	0.268	0.096	0.537	0.096
	*IAE*	405	0.305	0.115	0.681	0.088
	*PAE*	405	0.186	0.082	0.417	0.070
	*FEscale*	405	0.158	0.051	0.398	0.067
	*FEstructure*	405	3.852	1.218	8.882	1.282
	*Urban*	405	0.709	0.119	0.980	0.410
	*FEother*	405	0.220	0.170	1.399	0.043
	*LGS*	405	0.096	0.027	0.250	0.040
	*IS*	405	0.756	0.292	1.620	0.204
	*Open*	405	0.225	0.147	0.805	0.012
	*PI*	405	7.103	4.864	25.303	0.169
**Monocentric Structure**	*AE*	270	0.302	0.086	0.537	0.164
	*IAE*	270	0.348	0.107	0.681	0.185
	*PAE*	270	0.203	0.090	0.417	0.079
	*FEscale*	270	0.163	0.053	0.398	0.067
	*FEstructure*	270	3.944	1.350	8.882	1.282
	*Urban*	270	0.700	0.125	0.980	0.410
	*FEother*	270	0.245	0.200	1.399	0.057
	*LGS*	270	0.096	0.030	0.250	0.048
	*IS*	270	0.769	0.316	1.620	0.204
	*Open*	270	0.192	0.122	0.805	0.012
	*PI*	270	5.948	3.983	16.953	0.169
**Polycentric Structure**	*AE*	135	0.200	0.076	0.386	0.096
	*IAE*	135	0.224	0.085	0.431	0.088
	*PAE*	135	0.152	0.045	0.254	0.070
	*FEscale*	135	0.146	0.042	0.217	0.072
	*FEstructure*	135	3.667	0.872	6.022	1.591
	*Urban*	135	0.730	0.102	0.910	0.410
	*FEother*	135	0.170	0.055	0.272	0.043
	*LGS*	135	0.094	0.021	0.168	0.040
	*IS*	135	0.730	0.236	1.290	0.363
	*Open*	135	0.295	0.171	0.730	0.039
	*PI*	135	9.413	5.605	25.303	1.608

### Methodology

#### GMM.

According to the research samples and time, which is N = 27 and T = 16, we have chosen GMM (Hansen,1982) to verify the influence mechanism of the public services expenditure on the agglomeration effect in a region. Based on the general forms of GMM and the selection of variables, we have set up 6 models in the general equilibrium analysis, as follows:


$${AE}_{i,t}=\gamma +{\alpha_{1}AE}_{i,t-1}+{\beta FEscale}_{i,t}+{\delta_{i}Control\;variables}_{i,t}+u_{i}+\varepsilon_{i}$$ (1)

$${IAE}_{i,t}=\gamma +{\alpha_{1I}IAE}_{i,t-1}+{\beta FEscale}_{i,t}+{\delta_{i}Control\;variables}_{i,t}+u_{i}+\varepsilon_{i}\;$$ (2)

$${PAE}_{i,t}=\gamma +{\alpha_{1P}PAE}_{i,t-1}+{\beta FEscale}_{i,t}+{\delta_{i}Control\;variables}_{i,t}+u_{i}+\varepsilon_{i}$$ (3)

$${AE}_{i,t}=\gamma +{\alpha_{1}AE}_{i,t-1}+\beta {FEstructure}_{i,t}+{\delta_{i}Control\;variables}_{i,t}+u_{i}+\varepsilon_{i}$$ (4)

$$I{AE}_{i,t}=\gamma +{\alpha_{1I}IAE}_{i,t-1}+\beta {FEstructure}_{i,t}+{\delta_{i}Control\;variables}_{i,t}+u_{i}+\varepsilon_{i}$$ (5)

$${PAE}_{i,t}=\gamma +{\alpha_{1P}PAE}_{i,t-1}+{\beta FEscale}_{i,t}+{\delta_{i}Control\;variables}_{i,t}+u_{i}+\varepsilon_{i}$$ (6)

It should be noted that the existing mature spatial metrology models usually attempt to incorporate spatial or geographic factors into the models in the form of weights for explaining problems. However, the more commonly used spatial metrology models, such as spatial error model (SEM), spatial lag model (SAM) and spatial Durbin model (SDM), cannot divide the spatial structure very well and carry on the next interpretation. Therefore, we have chosen GMM as the empirical method for this study.

#### PTR.

In the theoretical deduction part, we have proposed that the change of urban size in a certain range may be a sudden factor affecting the agglomeration effect. Meanwhile, from the perspective of spatial structure, the level of the public services of second cities, likewise a sudden factor, would affect the agglomeration effect of the observed cities in the same region. Based on the above analysis, we have chosen the Panel Threshold Regression (PTR) to verify this fact. The result of dynamic threshold regression can test whether panel data has threshold effect and several threshold intervals. It can be divided into single threshold equation and multi-threshold equation, which are as follows:


$$Y_{i,t}=\mu_{i}++{\beta_{1}X}_{i,t}\cdot 1\;(q_{it}\leq \gamma_{1})+{{\beta_{2}X}_{i,t}\cdot 1\;\left(q_{it}>\gamma_{1}\right)+\delta_{i}^{\prime }Control\;variables}_{i,t}+u_{i}+\varepsilon_{i}$$

$$Y_{i,t}=\mu_{i}++{\beta_{1}X}_{i,t}\cdot 1\;(q_{it}\leq \gamma_{1})+{{\beta_{2}X}_{i,t}\cdot 1\;\left(\gamma_{1}{<q}_{it}\leq \gamma_{2}\right)+{\beta_{3}X}_{i,t}\cdot 1\;\left(q_{it}>\gamma_{2}\right)+\delta_{i}^{\prime }Control\;variables}_{i,t}+u_{i}+\varepsilon_{i}$$

when the threshold value is single, the threshold value of threshold regression is $\gamma_{1}$; when the threshold values are two, the threshold values of threshold regression are $\gamma_{1}$ and $\gamma_{2}$ ($\gamma_{1}<\gamma_{2}$). The general information on the method is presented in the supplementary material (S1 Appendix).

## Empirical results and discussion

### Stationarity tests

#### Unit root tests.

The ways of unit root tests are included IPS and HT which are applicable to short panel data as usual. Table 5 is shown the results of unit root tests, which are represented that *IAE* has passed the unit root tests directly, and the first-order differences of the rest of the variables have passed the unit root tests because the P-value is less 0.000.

**Table 5:** Unit root tests.

Variable	IPS	HT	Unit root
***AE***	-0.891	-0.887	**NO**
	(0.187)	(0.187)	
***D.AE***	-8.461	-24.174	YES
	(0.000)	(0.000)	
***IAE***	-2.366	-10.421	YES
	(0.009)	(0.000)	
***PAE***	4.366	0.939	**NO**
	(1.000)	(1.000)	
***D.PAE***	-8.269	-20.904	YES
	(0.000)	(0.000)	
***FEscale***	-0.637	-5.321	**NO**
	(0.262)	(0.000)	
***D.FEscale***	-8.363	-25.167	YES
	(0.000)	(0.000)	
***FEstructure***	-0.071	-5.237	**NO**
	(0.472)	(0.000)	
***D.FEstructure***	-8.421	-26.813	YES
	(0.000)	(0.000)	
***LGS***	-0.488	-5.928	**NO**
	(0.313)	(0.000)	
***D.LGS***	-5.266	-25.006	YES
	(0.000)	(0.000)	
***IS***	2.132	2.270	**NO**
	(0.984)	(0.988)	
***D.IS***	-6.702	-16.604	YES
	(0.000)	(0.000)	
***Open***	-1.229	-2.434	**NO**
	(0.110)	(0.008)	
***D. Open***	-8.875	-19.337	YES
	(0.000)	(0.000)	
***CS***	2.713	2.202	**NO**
	(0.997)	(0.986)	
***D.CS***	-7.447	-25.391	YES
	(0.000)	(0.000)	

Note: In this table, HT test (Harris-Tzavalis) includes the statistic and P-value, and IPS test (Im-Pesaran-Shin) includes the Z-value and the P-value.

#### Cointegration tests.

After the unit root tests, we prefer to use the original sequence for regression so that the cointegration tests are necessary. The methods of cointegration tests are Kao test and Pedroni test [78–80]. Table 6 is shown the results of cointegration tests, which represents that Model (1) – (6) are passed the tests through the t-value and the corresponding P-value.

**Table 6:** Cointegration tests.

Variable Relationship	Kao Test	Pedroni Test	
**ADF-t**	**MPP-t**	**ADF-t**
**AE-FEscale**	1.296	6.345	-3.350
	(0.098)	(0.000)	(0.000)
**IAE-FEstructure**	-5.117	5.023	-13.427
	(0.000)	(0.000)	(0.000)
**PAE-FEstructure**	1.216	6.487	-1.749
	(0.112)	(0.000)	(0.040)
**AE-FEstructure**	1.832	6.446	-4.104
	(0.034)	(0.000)	(0.000)
**IAE-FEstructure**	-3.922	5.142	-11.878
	(0.000)	(0.000)	(0.000)
**PAE-FEstructure**	1.777	6.550	-3.178
	(0.038)	(0.000)	(0.000)

Note: In this table, it is chosen ADF-t (Augmented Dickey-Fuller t) of the Kao test to be the part of the results of the cointegration tests. Meanwhile, it is chosen MPP-t (Modified Phillips-Perron t) and ADF-t (Augmented Dickey-Fuller t) of the Pedroni test to be the part of the results of the cointegration tests.

### Empirical results of the impact of public services expenditure on agglomeration effect

In general equilibrium analysis, we have used 2S-GMM to regress and obtained the regression results, which is shown in Table 7. In fact, before we decide to use the 2S-GMM model, it is necessary to conduct the comparative experiments of panel regression analysis including OLS, FE, RE and D-GMM, and the results of the experiments are shown in S13 Table (S1 Appendix). Even the 2S-GMM has been selected, we still need to make the necessary tests, including Bond tests which can test whether there is a first and second order autocorrelation of disturbance, Sargan tests and Wald tests which can test whether there is over-recognition of instrumental variables. The results have shown that the option of regression model is appropriate for Model (1) - Model (6).

**Table 7:** Empirical results of general equilibrium.

Variable	Model (1)	Model (2)	Model (3)	Model (4)	Model (5)	Model (6)
***L.AE***	0.734***			0.861***		
	(0.020)			(0.024)		
***L.IAE***		0.477***			0.777***	
		(0.019)			(0.014)	
***L.PAE***			0.952***			0.992***
			(0.008)			(0.010)
***FEscale***	0.311***	0.954***	0.073***			
	(0.048)	(0.048)	(0.004)			
***FEstructure***				0.006***	-0.008***	0.002***
				(0.001)	(0.001)	(0.000)
***LGS***	-0.129***	0.140***	-0.038***	-0.126***	0.280***	-0.048***
	(0.043)	(0.034)	(0.005)	(0.029)	(0.040)	(0.007)
***IS***	0.012***	0.022***	0.006***	-0.002	0.025***	0.004***
	(0.002)	(0.007)	(0.001)	(0.004)	(0.005)	(0.001)
***Open***	-0.030***	-0.098***	-0.012***	-0.033***	-0.075***	-0.015***
	(0.004)	(0.015)	(0.002)	(0.007)	(0.017)	(0.002)
***PI***	0.003***	-0.010***	0.001***	0.003***	-0.006***	0.002***
	(0.000)	(0.001)	(0.000)	(0.001)	(0.001)	(0.000)
***Constant***	0.011***	0.070***	-0.005**	0.010	0.113***	-0.008**
	(0.003)	(0.014)	(0.002)	(0.010)	(0.010)	(0.003)
***AR(1)***	0.0214	0.0180	0.0062	0.0179	0.0153	0.0063
***AR(2)***	0.4811	0.2318	0.2229	0.0921	0.3429	0.3400
***Sargan***	0.9880	0.9623	0.9863	0.9956	0.9682	0.9759
***Hanson***						
***Wald Test***	0.0000	0.0000	0.0000	0.0000	0.0000	0.0000

Note: Standard errors in parentheses. *** p < 0.01, ** p < 0.05, * p < 0.1.

The scale of public services expenditure has a positive influence on the agglomeration effect of provincial capital cities, which represents that is effective to enhance the agglomeration effect of a city by expanding persistently the scale of public services expenditure. We can find that the increase of the scale of public services has a strong effect on the agglomeration effect *(AE, IAE and PAE)*. However, the impact of public services expenditure structure on agglomeration economy is different. The impact of the structure of public services expenditure on the overall agglomeration effect and population agglomeration are positive, while opposite to the industrial agglomeration. It is noteworthy that the industrial agglomeration effect may pay more attention to the scale of public services expenditure than the population agglomeration effect, but be not sensitive to the change of expenditure structure. The reason could be that the industries only pay more attention to the overall expenditure behavior of local governments when they make a decision about the local settlement in a long-term. Besides, the industries could focus more on the expenditure directly related to its own profits, such as the expenditure of social security and medical security, which we have classified the types of the transfer expenditure, are directly related to the cost of local labor employed by enterprises resulted in determining the negative effect of expenditure structure on industrial agglomeration directly.

Among the control variables, the tax scale of local government *(LGS)* has a negative effect on the overall agglomeration effect and population agglomeration effect because of the obvious tax burden attached to residents., while a positive effect on industrial agglomeration effect because the tax incentives for industry from local governments are hidden. The influences of industry structure *(IS)* on the overall agglomeration effect and population agglomeration effect are positive but opposite to the industrial agglomeration effect. The influences of trade openness *(Open)* on agglomeration effect are significantly negative.

### Threshold regression tests

**Fig 3:**
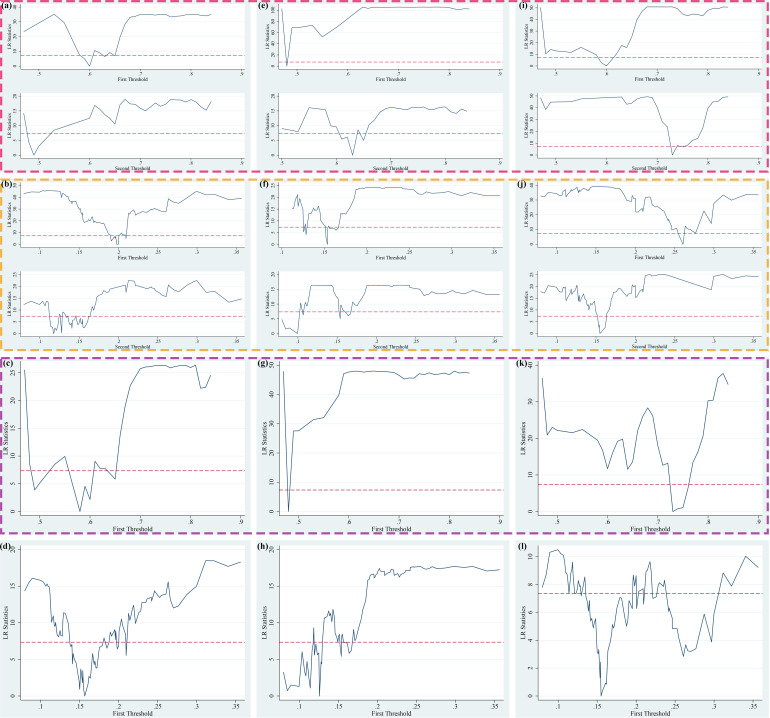
Threshold estimates of public services expenditure on agglomeration effects under the general equilibrium (PTR Model 1–12 corresponding to (a) – (l)).

Based on three explained variables (*AE, IAE and PAE*), two explanatory variables (*FEscale and FEstructure*) and two threshold variables (*Urban and FEother*), there are 36 threshold panel models constructed to test the existence of threshold values in the theoretical framework. With two threshold variables installed in advance, it can be found the threshold numbers, threshold estimates and confidence intervals for each model, expect for Model 8, Model 12, Model 20, Model 24, Model 25, Model 27, and Model 32. The testing process of threshold effect is shown in S14 Table, S18 Table and S22 Table (S1 Appendix). We have further drawn the LR diagram of the 95% confidence interval for *Urban* and *FEother* corresponding threshold values as shown in Fig 3–Fig 5, in which the horizontal axis represents the threshold variables, the vertical axis represents the LR values, and the dotted lines represent the 95% confidence interval reference lines. The lowest points of the curve crossing the reference lines of the confidence interval represent the corresponding threshold values, which are consistent with the threshold value obtained in Table 8. The results of threshold values are true and valid. It should be noted that we also summarize the LR statistical graph of the model without threshold to Fig 3–Fig 5 in order to be consistent with the established model.

**Fig 4:**
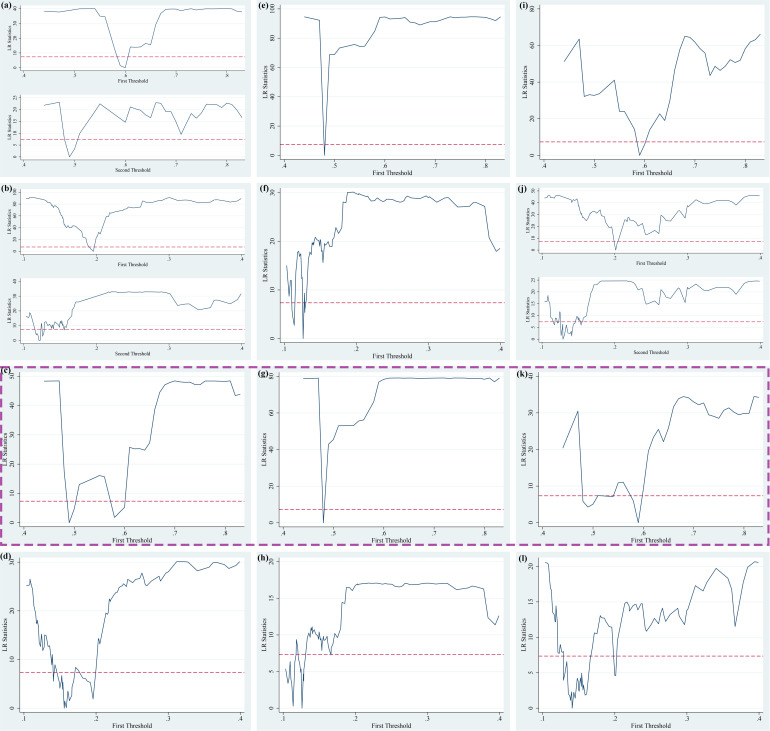
Threshold estimates of public services expenditure on agglomeration effects under the monocentric structure (PTR Model 13–24 corresponding to (a) – (l)).

**Fig 5:**
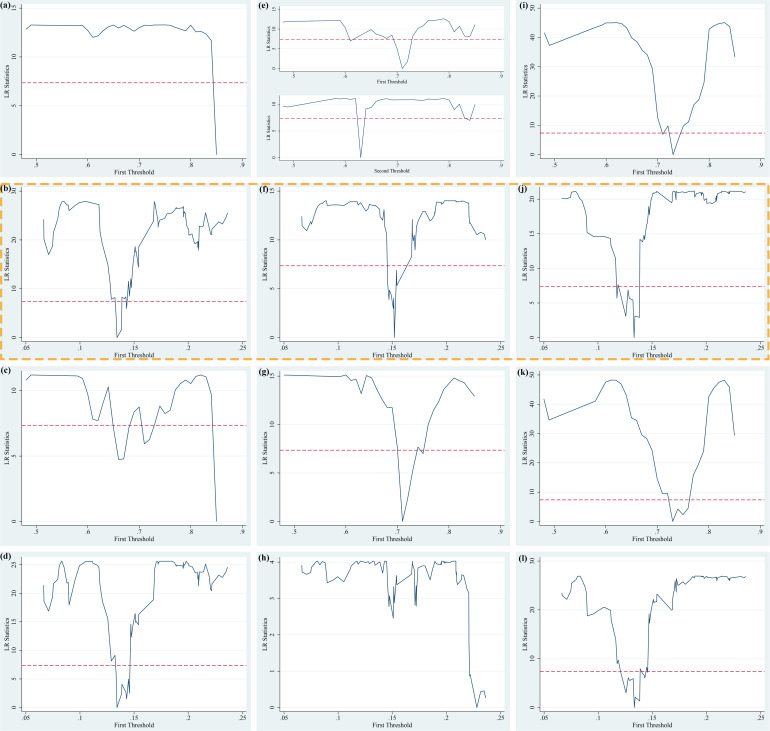
Threshold estimates of public services expenditure on agglomeration effects under the polycentric structure (PTR Model 25 - 36 corresponding to (a) – (l)).

**Table 8:** The tests of threshold effect.

Type	Explained Variable	Explaining Variable	Threshold Variable	PTR Model	Threshold Quantity	Threshold Value	
$\gamma_{1}$	$\gamma_{2}$
**GE**	AE	FEscale	Urban	1	2	0.490	0.600
			FEother	2	2	0.118	0.198
		FEstructure	Urban	3	1	0.580	***/***
			FEother	4	1	0.157	***/***
	IAE	FEscale	Urban	5	2	0.480	0.630
			FEother	6	2	0.100	0.154
		FEstructure	Urban	7	1	0.480	***/***
			FEother	8	0	***/***	***/***
	PAE	FEscale	Urban	9	2	0.600	0.730
			FEother	10	2	0.155	0.261
		FEstructure	Urban	11	1	0.730	***/***
			FEother	12	0	***/***	***/***
**MS**	AE	FEscale	Urban	13	2	0.490	0.600
			FEother	14	2	0.121	0.196
		FEstructure	Urban	15	1	0.490	***/***
			FEother	16	1	0.156	***/***
	IAE	FEscale	Urban	17	1	0.480	***/***
			FEother	18	1	0.126	***/***
		FEstructure	Urban	19	1	0.480	***/***
			FEother	20	0	***/***	***/***
	PAE	FEscale	Urban	21	1	0.590	***/***
			FEother	22	2	0.129	0.201
		FEstructure	Urban	23	1	0.590	***/***
			FEother	24	0	***/***	***/***
**PS**	AE	FEscale	Urban	25	0	***/***	***/***
			FEother	26	1	0.134	***/***
		FEstructure	Urban	27	0	***/***	***/***
			FEother	28	1	0.134	***/***
	IAE	FEscale	Urban	29	2	0.630	0.710
			FEother	30	1	0.152	***/***
		FEstructure	Urban	31	1	0.710	***/***
			FEother	32	0	***/***	***/***
	PAE	FEscale	Urban	33	1	0.730	***/***
			FEother	34	1	0.133	***/***
		FEstructure	Urban	35	1	0.730	***/***
			FEother	36	1	0.133	***/***

Note: In this table, the number of thresholds and the corresponding threshold values for each model is represented.

From Fig 3 corresponding to the two threshold variables under the general equilibrium, we can find that based on the influences of the scale of public services expenditure, the threshold variable *(Urban)* in Model 1, Model 5 and Model 9 all have 2 threshold values. Based on the influences of the scale of public services expenditure, the threshold variable *(Feother)* in Model 2, Model 6 and Model 10 all have two threshold values. Based on the influences of the structure of public services expenditure, the threshold variable *(Urban)* in Model 3, Model 7 and Model 11 all have one threshold value. From Fig 4 corresponding to the two threshold variables under the monocentric structure, we can find that under the influences of the structure of public services expenditure, the threshold variable *(Urban)* in Model 15, Model 19 and Model 23 all have one threshold value. From Fig 5 corresponding to the two threshold variables under the polycentric structure, we can find that based on the influences of the scale of public services expenditure, the threshold variable *(FEother)* in Model 26, Model 30 and Model 34 all have one threshold value. In the subsequent empirical analysis of threshold regression, we have analyzed the results of the regression models with commonalities in the above three figures, and put the threshold regression results of 36 models in S15 Table – S25 Table (S1 Appendix).

### Threshold estimates of the general equilibrium

In the analysis of general equilibrium, from the Table 9, we can find that the double threshold values of *Urban*, which is the threshold variable for the impact of public services expenditure scale on *AE* and *PAE*, is relatively close. Respectively, the threshold values are 0.490 and 0.600 for *AE*; 0.480 and 0.630 for *IAE.* The threshold values for *PAE* are larger, corresponding to 0.600 and 0.730. It can be represented that the population agglomeration effect of provincial capital cities is more sensitive to the change of urbanization level. After crossing the first threshold and the second threshold, the promoting effect of the scale of public services expenditure on *AE* and *PAE* increases significantly. It is further explained that there is a threshold effect based on urban size between the scale of public services expenditure and agglomeration effect, and the continuous expansion of urban size is conducive to the promotion of the scale of fiscal expenditure to agglomeration effect. However, the threshold effect *(Urban)* of public services expenditure on *IAE* is inverted U-shaped. The scale of public services expenditure has the largest promoting effect on *IAE* when the threshold value is [0.480, 0.630], while there is a negative effect when the threshold value is less than 0.480.

**Table 9:** Threshold regression results in the general equilibrium analysis.

Variable	M1-AE	M5-IAE	M9-PAE
***LGS***	-0.161***	0.262***	-0.096***
	(0.054)	(0.073)	(0.033)
***IS***	-0.027***	0.002	-0.005
	(0.007)	(0.010)	(0.004)
***Open***	-0.004	0.011	-0.009
	(0.012)	(0.017)	(0.008)
***PI***	-0.001	0.003**	0.007***
	(0.001)	(0.001)	(0.001)
***FEscale*** $(Urban\leq \gamma_{1})$	-0.006	-0.257***	0.257***
	(0.071)	(0.097)	(0.038)
***FEscale*** ${(\gamma }_{1}<Urban\leq \gamma_{2})$	0.173***	0.215**	0.361***
	(0.062)	(0.084)	(0.039)
***FEscale*** $(Urban>\gamma_{2})$	0.349***	0.117	0.442***
	(0.064)	(0.086)	(0.034)
***Constant***	0.261***	0.240***	0.093**
	(0.015)	(0.021)	(0.009)
***F***	33.12	18.23	100.54
	(0.0000)	(0.0000)	(0.0000)
***R2***	0.3846	0.2559	0.6548

Note: Standard errors in parentheses. *** p < 0.01, ** p < 0.05, * p < 0.1.

From the Table 10 we can find that the double threshold values of *FEother*, that is the threshold variable for the impact of public services expenditure scale on *AE* and *PAE*, is relatively close. Respectively, the threshold values are 0.118 and 0.198 for *AE*; 0.100 and 0.154 for *IAE*. The threshold values for *PAE* are larger, corresponding to 0.155 and 0.261. It can be represented that the population agglomeration effect of provincial capital cities is more sensitive to the changes of public services of second cities around the region. After crossing the first threshold and the second threshold, the promoting effects of the scale of public services expenditure on *AE*, *IAE* and *PAE* increase significantly. It is further explained that there is a threshold effect based on *FEother* between the scale of public services expenditure and agglomeration effect, and the continuous improvement of the scale of public services of the second city in the region would promote the improvement of the scale of fiscal expenditure to the agglomeration effect. It is worth noting that the influence of fiscal expenditure scale on industrial agglomeration effect is negative when the threshold values of *FEother* is less than 0.100.

**Table 10:** Threshold regression result in the general equilibrium analysis.

Variable	M2-AE	M6-IAE	M10-PAE
***LGS***	-0.316***	0.118	-0.191***
	(0.058)	(0.079)	(0.036)
***IS***	-0.043***	-0.009	-0.025***
	(0.007)	(0.010)	(0.004)
***Open***	0.008	0.003	-0.001
	(0.013)	(0.018)	(0.008)
***PI***	-0.001	-0.001	0.007***
	(0.001)	(0.001)	(0.001)
***FEscale*** $(FEother\leq \gamma_{1})$	0.114*	-0.147	0.231***
	(0.066)	(0.107)	(0.040)
***FEscale*** ${(\gamma }_{1}<FEother\leq \gamma_{2})$	0.217***	0.165*	0.300***
	(0.065)	(0.089)	(0.042)
***FEscale*** $(FEother>\gamma_{2})$	0.382***	0.302***	0.421***
	(0.067)	(0.091)	(0.041)
***Constant***	0.297***	0.266***	0.125***
	(0.015)	(0.021)	(0.009)
***F***	26.74	10.84	86.86
	(0.0000)	(0.0000)	(0.0000)
***R2***	0.3354	0.1698	0.6210

Note: Standard errors in parentheses. *** p < 0.01, ** p < 0.05, * p < 0.1.

From the Table 11 we can find the single threshold value of *Urban*, which is the threshold variable for the impact of the structure of public services expenditure on *AE*, *IAE* and *PAE*. Respectively, the threshold value is 0.580 for *AE*; 0.480 for *IAE*; 0.730 for *PAE*. As *Urban* is less than the threshold value, the influences of the structure of public services expenditure on agglomeration effect are negative. After crossing the threshold value, the effect of public services expenditure on *AE* turns positive, while the effect on *IAE* and *PAE* effect has diminished and still negative.

**Table 11:** Threshold regression result in the general equilibrium analysis.

Variable	M3-AE	M7-IAE	M11-PAE
***LGS***	-0.110*	0.308***	0.026
	(0.061)	(0.079)	(0.039)
***IS***	-0.047***	-0.001	-0.016***
	(0.008)	(0.010)	(0.005)
***Open***	-0.003	0.033*	0.005
	(0.014)	(0.020)	(0.009)
***PI***	-0.001	0.002	0.008***
	(0.001)	(0.001)	(0.001)
***FEstructure*** $(Urban\leq \gamma_{1})$	-0.006***	-0.016***	-0.005***
	(0.002)	(0.003)	(0.001)
***FEstructure*** $(Urban>\gamma_{1})$	0.002	-8.24e-05	-0.002
	(0.001)	(0.002)	(0.001)
***Constant***	0.315***	0.259***	0.154***
	(0.014)	(0.019)	(0.009)
***F***	19.70	11.51	73.89
	(0.0000)	(0.0000)	(0.0000)
***R2***	0.2411	0.1565	0.5437

Note: Standard errors in parentheses. *** p < 0.01, ** p < 0.05, * p < 0.1.

To sum up, in the general equilibrium analysis, in the wake of expansion of urban scale, the impact of scale of public service expenditure on agglomeration effect and population agglomeration effect increases continuously, while the impact on industrial agglomeration effect firstly increases and then decreases. With the expansion of scale of the public services expenditure of the second largest city in the region the impact of scale of public service expenditure of provincial capital cities on agglomeration effect increase. Besides, in the wake of expansion of urban scale, the impact of structure of public service expenditure on agglomeration effect and population agglomeration effect increase, while the impact on industrial agglomeration effect decreases.

### Threshold estimates of the monocentric structure

In the analysis of monocentric structure, from the Table 12, we can find that the single threshold values of *Urban*, that is the threshold variable for the impact of structure of public services expenditure scale on *AE* and *IAE*, is relatively close. Respectively, the threshold values are 0.490 for *AE*; 0.480 for *IAE*. The threshold values for *PAE* are larger, corresponding to 0.590. It can be represented that the population agglomeration effect of provincial capital cities is more sensitive to the urban scale in the region. As Urban is less than the threshold value, the influences of the structure of public services expenditure on agglomeration effect are negative. After crossing the threshold value, the effect of public services expenditure on *AE* and *PAE* turns positive, while the effect on *IAE* is still negative and intensified.

**Table 12:** Threshold regression results of the monocentric structure.

Variable	M15-AE	M19-IAE	M23-PAE
***LGS***	0.040	0.370***	0.117**
	(0.077)	(0.087)	(0.047)
***IS***	-0.048***	-0.009	-0.017***
	(0.010)	(0.012)	(0.006)
***Open***	0.011	0.069***	-0.015
	(0.020)	(0.022)	(0.011)
***PI***	2.55e-05	0.001	0.013***
	(0.002)	(0.002)	(0.001)
***FEstructure*** $(Urban\leq \gamma_{1})$	-0.011***	-0.024***	-0.012***
	(0.003)	(0.004)	(0.002)
***FEstructure*** $(Urban>\gamma_{1})$	0.002	0.001	-0.004***
	(0.002)	(0.003)	(0.001)
***Constant***	0.333***	0.308***	0.154***
	(0.019)	(0.021)	(0.011)
***F***	13.11	16.11	63.69
	(0.0000)	(0.0000)	(0.0000)
***R2***	0.2422	0.2820	0.6084

Note: Standard errors in parentheses. *** p < 0.01, ** p < 0.05, * p < 0.1.

### Threshold estimates of the polycentric structure

In the analysis of the polycentric structure, from the Table 13, we can find that the single threshold values of *FEother*, that is the threshold variable for the impact of the scale of public services expenditure on *AE* and *PAE*, is relatively close. Respectively, the threshold values are 0.134 for *AE*; 0.133 for *PAE*. The threshold values for *IAE* are larger, corresponding to 0.152. And the influences of the scale of public services expenditure on agglomeration effect are positive. However, as *FEother* crosses the threshold value, the influence of the scale of public services expenditure on overall agglomeration effect has diminished from 0.251 to 0.084. As *FEother* crosses the threshold value, the influence of the scale of public services expenditure on population agglomeration effect has diminished from 0.211 to 0.131. After crossing the 0.152 of the threshold value, the effect on industrial agglomeration effect enlarges from 0.716 to 0.973.

**Table 13:** Threshold regression results of the polycentric structure.

Variable	M26-AE	M30-IAE	M34-PAE
***LGS***	-0.567***	0.043	-0.062
	(0.074)	(0.182)	(0.050)
***IS***	-0.052***	0.009	-0.024***
	(0.007)	(0.016)	(0.005)
***Open***	-0.013	-0.028	0.058***
	(0.015)	(0.033)	(0.010)
***PI***	0.001	-0.001	0.008***
	(0.001)	(0.002)	(0.001)
***FEscale*** $(FEother\leq \gamma_{1})$	0.251**	0.716***	0.211***
	(0.101)	(0.234)	(0.069)
***FEscale*** $(FEother>\gamma_{1})$	0.084	0.973***	0.131*
	(0.109)	(0.256)	(0.072)
***Constant***	0.263***	0.099**	0.065***
	(0.022)	(0.049)	(0.015)
***F***	37.64	5.40	56.31
	(0.0000)	(0.0000)	(0.0000)
***R2***	0.6530	0.2125	0.7379

Note: Standard errors in parentheses. *** p < 0.01, ** p < 0.05, * p < 0.1.

## Discussion

In conclusion, the following three conclusions are obtained:

(1) the scale of public service expenditure has a positive influence on agglomeration effect. The larger the scale of public service expenditure, the industrial agglomeration effect shows a significant positive effect. The structure of public service expenditure has a positive effect on the aggregate agglomeration effect and the population agglomeration effect, and a significant negative effect on the industrial agglomeration effect.
(2) in monocentric structure areas, the strongest agglomeration effect needs a matching interval between the public service expenditure and the scale of urban expansion of the core city. Meanwhile, the public services expenditure structure has different degrees of impact on agglomeration effect in the changes of urban scale. When urban scale exceeds the threshold level, the impacts on agglomeration effect and industrial agglomeration effect begin to increase and become positive, and the impacts on population agglomeration is improved.
(3) in polycentric structure areas, the contribution of public services expenditure of core city to the agglomeration effect is closely related to the public services expenditure of the other core city in a province. The public services expenditure structure has different degrees of impact on agglomeration effect in the changes of the scale of public services in the second central city. When the public service scale of the second central city exceeds the threshold level, the impacts on agglomeration effect on the agglomeration effect and population agglomeration effect is positive but shrink, while the impacts on industrial agglomeration effect is positive and increase.

The argumentation process and conclusions of this study may be influenced by factors such as the policy environment, and existing laws and regulations, which could limit the applicability of the findings. Based on the research, we will continue to study the mechanism of government agglomeration in the future, construct the spatial effect of government economic activities, and explore the close relationship between agglomeration and government economic activities.

## Supporting information

S1 Appendix (PDF)
