# Supplementary material for: Public services and agglomeration effect under spatial structure: Threshold verification on the provincial capital cities in China
Source: PLoS One. 2025 May 9;20(5):e0321466. doi: 10.1371/journal.pone.0321466 (PMC12063852; doi:10.1371/journal.pone.0321466)
Supplement: S1 Appendix — (PDF) [file pone.0321466.s001.pdf]

Supplemental Appendix

Table of contents

Section 1: Graphical abstract	3-4
S1 Fig	3
Section 2: The classifications of spatial structure	5
S2 Fig	5
Section 3: The relationship between AE and FE	6
S3 Fig	6
Section 4: The Data of all variables	7
S1 Table	7-8
S2 Table	8-9
S3 Table	9-10
S4 Table	10-11
S5 Table	11-12
S6 Table	12-13
S7 Table	13-15
S8 Table	15-16
S9 Table	16-17
S10 Table	17-18
S11 Table	18-19
Section 5: The Structure on fiscal expenditure	20
S12 Table	20
Section 6: Method	21-22
Section 7: Comparative experiment of empirical research	23
S13 Table	23-24

Section 8: Threshold tests	25
S14 Table	25-26
S15 Table	26-27
S16 Table	27-28
S17 Table	28
S18 Table	29-30
S19 Table	30-31
S20 Table	31
S21 Table	31-32
S22 Table	32-33
S23 Table	33
S24 Table	34-35
S25 Table	35-36
References	37

Section 1: Graphical Abstract.

Based on the literature analysis, theoretical and empirical verification, this paper studies the relationship between fiscal expenditure of the public services and agglomeration effect on the basis of provincial capital cities, which discussed whether the fiscal expenditure of the capital city has a sustainable effect on the economic development. We have built a general equilibrium model and two types spatial structures models of basic public service expenditure and the agglomeration effect, which we divided a region into monocentric structure and polycentric structure. We have chosen 27 provincial capital cities in China where are a political centre or an economic centre region in order to obtain the precise verification. The result of the research can help many regions with the similar situation to solve the problem of allocation of fiscal resources and fiscal sustainability. The graphical abstract (S1 Fig) of the work in the paper is as follows:

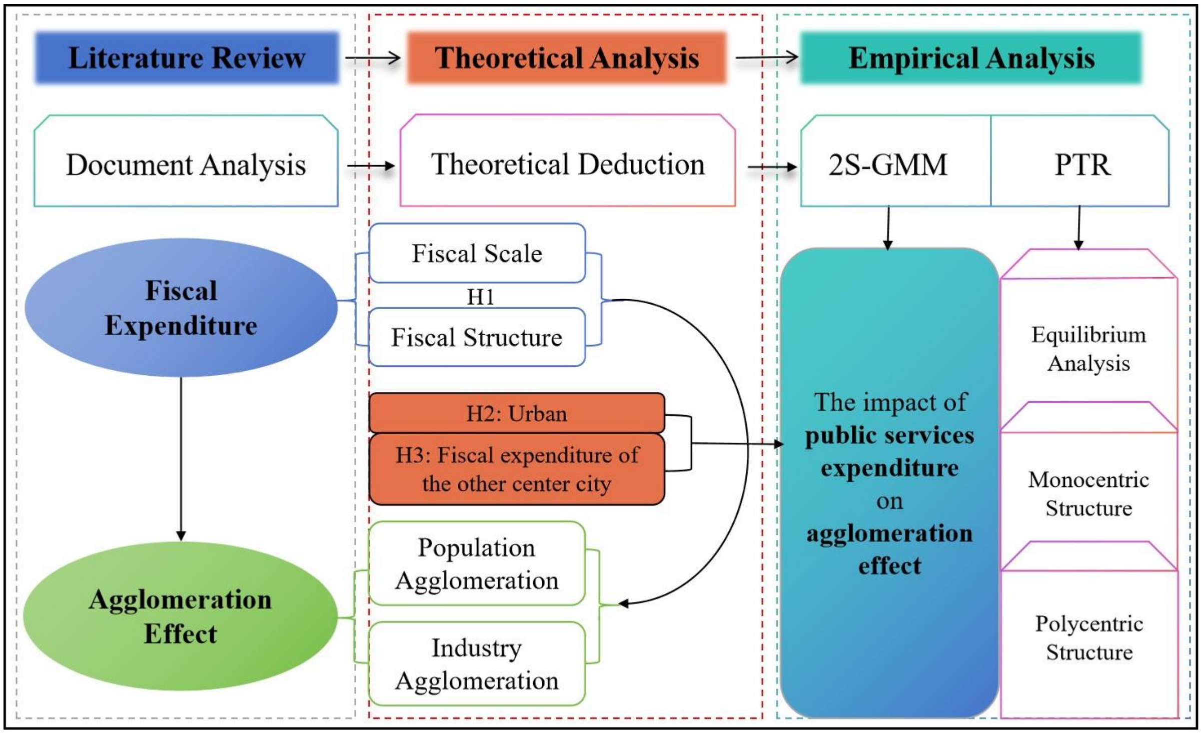

S1 Fig. Graphical abstract.

Local governments often face two pressures on their finances. On the one hand, they need to meet the growing demand for public services such as education, health care and infrastructure [S1]. On the other hand, fiscal revenue may be affected by multiple factors such as slowing economic growth, unreasonable tax structure or policy adjustments [S2-S3]. This fiscal dilemma does not exist in isolation, but is closely linked to economic development, social change, and policy choices. When local governments face fiscal difficulties, they often have to balance investment in public services against economic stagnation. By increasing investment in public services (such as education, health care, transportation, etc.), local governments can attract more talent, enterprises, and capital inflows, thereby driving economic growth and urban development. This agglomeration effect can not only bring direct economic benefits, but also improve the quality of life and competitiveness of the city [S4]. If local governments reduce public service investment due to fiscal constraints, it may lead to backward urban infrastructure and a decline in the level of public services, which will affect the attractiveness and competitiveness of the city. In the long run, this could lead to slower or even stagnant economic growth, creating a vicious circle.

Section 2. The classifications of spatial structure.

We have added indicators of economic primacy to support our division of spatial structure. The concept of primacy ratio is proposed to solve the problem of urban scale [S1]. The calculation equation is as follows:

$$P_r = \frac{P_1}{P_2}$$

P_1 represents the economic indicators of the NO.1 city in the region, and P_2 represents the economic indicators of the NO.2 city in the region. P_r represents primacy ratio. In this paper, we set cities with P_r greater than 1 as monocentric structure, while cities with P_r less than 1 as polycentric structure. The result of the division is consistent with the division in our manuscript, which is shown in S2 Fig (S1 Appendix).

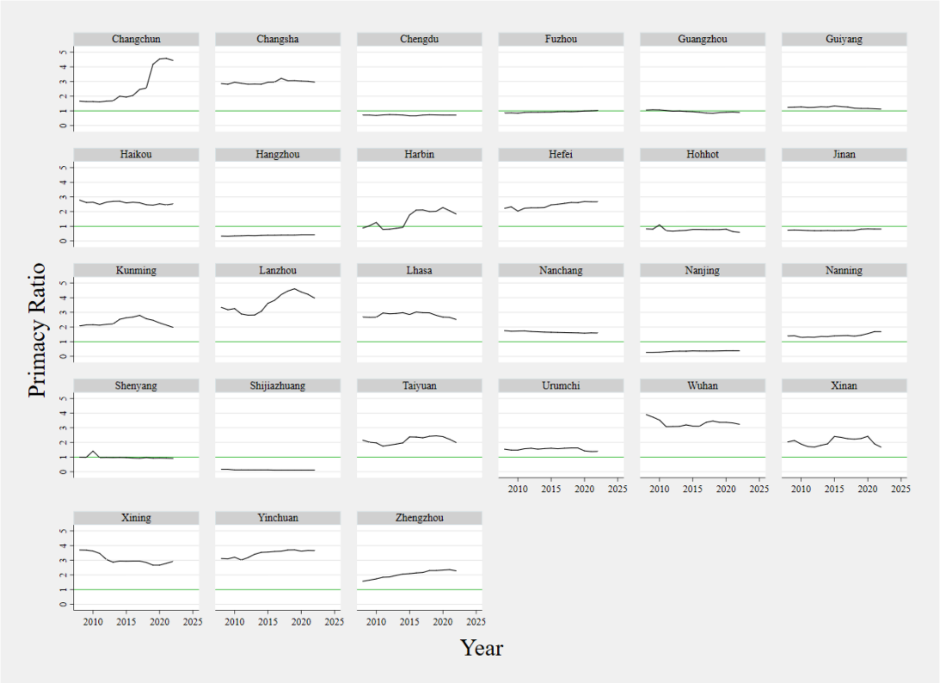

S2 Fig. The primacy ratio of the provincial cities in China.

Section 3 The relationship between AE and FE

By observing the correlation coefficient of AE and FE and the correlation coefficient of AE and GDP growth rate in China., which is shown in S13 Fig. We can find that the correlation between agglomeration effect (AE) and fiscal expenditure (FE) is closely positive. Meanwhile, the correlation between agglomeration effect and GDP growth rate is unstable. This explains the relationship between agglomeration economy, economic growth and fiscal expenditure.

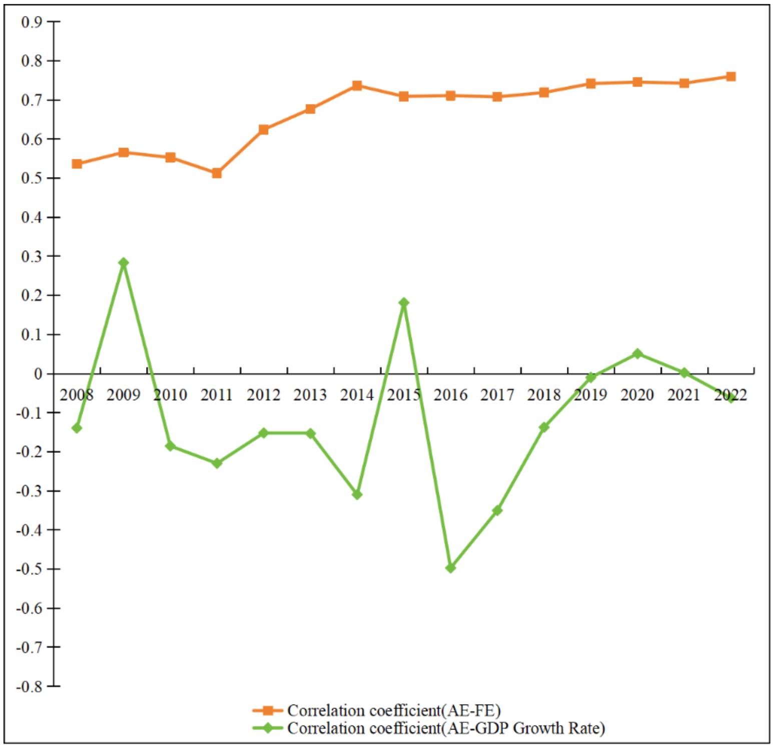

S3 Fig. The correlation coefficient of AE and FE and the correlation coefficient of AE and GDP growth rate in China.

Section 4. The Data of all variables.

S1 Table – S11 Table have shown the data of all variables, which are included the three explained variables, two explaining variables, two threshold variables and four control variables.

S1 Table. The agglomeration effect of 27 provincial capital cities in China.

provincial capital cities	2008	2009	2010	2011	2012	2013	2014	2015	2016	2017	2018	2019	2020	2021	2022
Shijiazhuang	0.17	0.17	0.14	0.14	0.14	0.14	0.14	0.14	0.14	0.15	0.15	0.17	0.16	0.16	0.17
Taiyuan	0.21	0.20	0.19	0.18	0.19	0.19	0.19	0.21	0.21	0.21	0.22	0.24	0.24	0.23	0.22
Hohhot	0.12	0.11	0.16	0.10	0.10	0.10	0.11	0.12	0.12	0.15	0.15	0.16	0.16	0.15	0.14
Shenyang	0.22	0.21	0.27	0.18	0.18	0.18	0.18	0.18	0.24	0.24	0.25	0.26	0.26	0.26	0.27
Changchun	0.34	0.34	0.34	0.34	0.34	0.34	0.34	0.34	0.34	0.36	0.37	0.50	0.54	0.54	0.52
Harbin	0.23	0.26	0.35	0.23	0.23	0.25	0.25	0.27	0.28	0.30	0.31	0.38	0.37	0.36	0.35
Nanjing	0.13	0.12	0.12	0.13	0.14	0.14	0.14	0.14	0.14	0.14	0.14	0.14	0.14	0.14	0.14
Hangzhou	0.23	0.22	0.22	0.22	0.23	0.22	0.24	0.24	0.25	0.25	0.25	0.25	0.25	0.25	0.24
Hefei	0.19	0.21	0.22	0.24	0.24	0.25	0.25	0.26	0.27	0.27	0.29	0.25	0.26	0.27	0.27
Fuzhou	0.22	0.22	0.21	0.22	0.22	0.22	0.22	0.22	0.23	0.23	0.24	0.22	0.23	0.23	0.23
Nanchang	0.25	0.23	0.23	0.23	0.22	0.22	0.22	0.23	0.22	0.23	0.23	0.22	0.23	0.23	0.22
Jinan	0.10	0.10	0.10	0.10	0.10	0.10	0.10	0.10	0.10	0.10	0.10	0.13	0.14	0.14	0.14
Zhengzhou	0.16	0.17	0.17	0.18	0.19	0.19	0.19	0.20	0.20	0.21	0.22	0.21	0.22	0.22	0.21
Wuhan	0.36	0.37	0.34	0.34	0.35	0.35	0.37	0.36	0.35	0.37	0.38	0.35	0.36	0.35	0.35
Changsha	0.28	0.28	0.28	0.28	0.28	0.28	0.28	0.29	0.29	0.30	0.29	0.29	0.29	0.29	0.29
Guangzhou	0.23	0.23	0.23	0.23	0.23	0.24	0.24	0.24	0.23	0.22	0.22	0.22	0.23	0.23	0.22
Nanning	0.17	0.18	0.18	0.17	0.18	0.18	0.18	0.19	0.19	0.21	0.20	0.21	0.21	0.21	0.20
Haikou	0.30	0.29	0.31	0.28	0.30	0.31	0.31	0.31	0.32	0.32	0.32	0.32	0.32	0.32	0.31
Chengdu	0.34	0.33	0.32	0.35	0.36	0.36	0.36	0.35	0.36	0.38	0.39	0.36	0.37	0.37	0.37
Guiyang	0.2	0.2	0.2	0.2	0.2	0.2	0.2	0.2	0.2	0.2	0.2	0.2	0.2	0.2	0.2

	4	4	4	4	4	5	5	8	7	6	5	4	4	4	4
Kunming	0.29	0.30	0.30	0.29	0.30	0.30	0.30	0.32	0.32	0.33	0.34	0.28	0.27	0.27	0.26
Lhasa	0.35	0.35	0.35	0.37	0.37	0.38	0.38	0.37	0.39	0.38	0.38	0.36	0.36	0.36	0.35
Xinan	0.34	0.33	0.32	0.30	0.30	0.31	0.32	0.33	0.33	0.34	0.35	0.36	0.38	0.36	0.35
Lanzhou	0.27	0.28	0.27	0.28	0.29	0.29	0.29	0.31	0.31	0.33	0.32	0.33	0.32	0.32	0.30
Xining	0.47	0.46	0.47	0.46	0.44	0.46	0.47	0.47	0.49	0.49	0.45	0.45	0.46	0.46	0.46
Yinchuan	0.52	0.46	0.45	0.45	0.45	0.46	0.46	0.45	0.45	0.47	0.48	0.51	0.50	0.50	0.50
Urumchi	0.24	0.25	0.24	0.26	0.27	0.26	0.27	0.28	0.25	0.25	0.25	0.23	0.24	0.23	0.22

S2 Table. The industrial agglomeration effect of 27 provincial capital cities in China.

provincial capital cities	2008	2009	2010	2011	2012	2013	2014	2015	2016	2017	2018	2019	2020	2021	2022
Urumchi	0.2753	0.3007	0.2946	0.2917	0.3029	0.2954	0.2877	0.3017	0.3024	0.2900	0.2926	0.2865	0.2801	0.2689	0.2535
Lanzhou	0.3109	0.3165	0.3130	0.3171	0.3293	0.3295	0.3335	0.3546	0.3492	0.3640	0.3644	0.3653	0.3608	0.3564	0.3385
Nanjing	0.1305	0.1287	0.1250	0.1302	0.1390	0.1406	0.1404	0.1410	0.1398	0.1400	0.2462	0.1441	0.1479	0.1438	0.1407
Nanning	0.2073	0.2142	0.2084	0.2108	0.2174	0.2248	0.2244	0.2270	0.2261	0.2279	0.2246	0.2241	0.2269	0.2178	0.2096
Nanchang	0.2885	0.2662	0.2534	0.2506	0.2497	0.2503	0.2494	0.2541	0.2515	0.2618	0.2440	0.2372	0.2341	0.2332	0.2348
Hefei	0.2092	0.2330	0.2417	0.2378	0.2411	0.2652	0.2423	0.2515	0.2521	0.2562	0.2405	0.2666	0.2736	0.2793	0.2801
Hohhot	0.1257	0.1140	0.1640	0.1608	0.1584	0.1054	0.1664	0.1743	0.1751	0.1759	0.1733	0.1744	0.1734	0.1658	0.1545
Harbin	0.3432	0.3982	0.3588	0.4607	0.4545	0.3986	0.4972	0.5657	0.5917	0.6062	0.5866	0.4487	0.4452	0.4138	0.3920
Taiyuan	0.2113	0.2258	0.2081	0.1985	0.2061	0.2103	0.2194	0.2428	0.2600	0.2427	0.2526	0.2460	0.2467	0.2383	0.2273
Guangzhou	0.2314	0.2383	0.2381	0.2395	0.2456	0.2548	0.2531	0.2498	0.2463	0.2416	0.2355	0.2263	0.2333	0.2339	0.2304
Chengdu	0.3581	0.3555	0.3582	0.3640	0.3749	0.3834	0.3824	0.3908	0.4000	0.3980	0.3852	0.3947	0.3973	0.4012	0.3983
Lhasa	0.4055	0.3941	0.3977	0.4123	0.4174	0.4236	0.4210	0.4105	0.4126	0.4092	0.4073	0.3833	0.3743	0.3744	0.3692
Kunming	0.2992	0.3067	0.3010	0.2924	0.3016	0.2960	0.2929	0.2936	0.2899	0.2878	0.2712	0.3074	0.3066	0.2960	0.2892
Hangzhou	0.2277	0.2262	0.2200	0.2235	0.2303	0.2268	0.2331	0.2340	0.2369	0.2426	0.2357	0.2497	0.2527	0.2493	0.2442
Wuhan	0.3995	0.4052	0.3868	0.3753	0.3970	0.4041	0.4016	0.4019	0.4001	0.3969	0.4044	0.3771	0.3870	0.3809	0.3773
Shenyang	0.3024	0.3049	0.2844	0.3844	0.3935	0.2756	0.3758	0.3822	0.2800	0.2828	0.4309	0.2721	0.2746	0.2756	0.2790

Jinan	0.0884	0.1035	0.1039	0.1006	0.0997	0.0990	0.1003	0.0999	0.0986	0.1015	0.1710	0.1380	0.1443	0.1430	0.1431
Haikou	0.3933	0.3981	0.3917	0.3885	0.3821	0.3904	0.3896	0.3754	0.3913	0.3843	0.3751	0.3801	0.3912	0.3741	0.3769
Shijiazhuang	0.1787	0.1897	0.1700	0.1963	0.2008	0.1764	0.2124	0.2123	0.2118	0.2174	0.3298	0.1697	0.1682	0.1646	0.1723
Fuzhou	0.2186	0.2171	0.2171	0.2146	0.2160	0.2169	0.2171	0.2168	0.2209	0.2227	0.2209	0.2248	0.2298	0.2328	0.2323
Xining	0.5080	0.5775	0.4331	0.4646	0.4403	0.4594	0.4712	0.4736	0.4791	0.4946	0.4899	0.4792	0.4924	0.4976	0.4896
Xinan	0.3230	0.3600	0.3485	0.3359	0.3254	0.3231	0.3322	0.3430	0.3473	0.3643	0.3659	0.3799	0.4059	0.3789	0.3697
Guiyang	0.2742	0.2553	0.2677	0.2686	0.2769	0.2872	0.3028	0.3103	0.3042	0.2930	0.2762	0.2677	0.2704	0.2680	0.2726
Zhengzhou	0.1847	0.1931	0.1963	0.2111	0.2130	0.2155	0.2168	0.2180	0.2206	0.2249	0.2306	0.2306	0.2353	0.2319	0.2296
Yinchuan	0.5566	0.4952	0.4947	0.5188	0.5232	0.4806	0.5366	0.5377	0.5327	0.5282	0.5289	0.5283	0.5316	0.5320	0.5242
Changchun	0.4256	0.4218	0.4039	0.5601	0.5532	0.4072	0.5798	0.5929	0.6029	0.6325	0.6810	0.5322	0.5674	0.5632	0.5442
Changsha	0.3092	0.3215	0.3169	0.3181	0.3200	0.3206	0.3144	0.3176	0.3236	0.3287	0.3205	0.3106	0.3122	0.3077	0.3067

S3 Table. The population agglomeration effect of 27 provincial capital cities in China.

provincial capital cities	2008	2009	2010	2011	2012	2013	2014	2015	2016	2017	2018	2019	2020	2021	2022
Shijiazhuang	0.138	0.140	0.141	0.142	0.143	0.144	0.145	0.146	0.146	0.147	0.147	0.148	0.151	0.150	0.151
Taiyuan	0.102	0.102	0.118	0.121	0.123	0.129	0.131	0.134	0.138	0.142	0.146	0.150	0.152	0.155	0.156
Hohhot	0.113	0.115	0.116	0.118	0.121	0.126	0.129	0.132	0.134	0.137	0.139	0.141	0.144	0.146	0.148
Shenyang	0.165	0.181	0.185	0.187	0.187	0.189	0.190	0.191	0.199	0.202	0.206	0.209	0.213	0.216	0.218
Changchun	0.282	0.281	0.279	0.279	0.279	0.287	0.289	0.291	0.296	0.300	0.304	0.308	0.378	0.383	0.386
Harbin	0.272	0.275	0.277	0.276	0.274	0.285	0.288	0.292	0.315	0.322	0.326	0.331	0.316	0.316	0.319
Nanjing	0.098	0.099	0.102	0.103	0.103	0.100	0.099	0.099	0.099	0.099	0.100	0.100	0.110	0.111	0.111
Hangzhou	0.157	0.154	0.160	0.160	0.161	0.153	0.172	0.173	0.175	0.177	0.179	0.182	0.185	0.187	0.188
Hefei	0.082	0.083	0.125	0.126	0.126	0.127	0.128	0.138	0.141	0.144	0.147	0.150	0.153	0.155	0.157
Fuzhou	0.188	0.187	0.193	0.198	0.199	0.196	0.196	0.196	0.196	0.198	0.199	0.199	0.200	0.192	0.202
Nanchang	0.105	0.105	0.113	0.115	0.117	0.120	0.122	0.125	0.128	0.131	0.133	0.136	0.138	0.143	0.144
Jinan	0.070	0.071	0.071	0.071	0.072	0.072	0.072	0.073	0.073	0.073	0.074	0.088	0.091	0.092	0.093
Zhengzhou	0.079	0.079	0.092	0.097	0.101	0.103	0.107	0.099	0.114	0.118	0.122	0.125	0.127	0.129	0.130

Wuhan	0.157	0.159	0.171	0.174	0.175	0.176	0.178	0.181	0.183	0.185	0.187	0.189	0.217	0.234	0.235
Changsha	0.103	0.104	0.107	0.112	0.115	0.119	0.123	0.125	0.130	0.136	0.140	0.145	0.151	0.155	0.158
Guangzhou	0.113	0.117	0.122	0.128	0.134	0.131	0.133	0.137	0.141	0.144	0.146	0.147	0.148	0.148	0.148
Nanning	0.144	0.144	0.145	0.148	0.151	0.153	0.156	0.159	0.162	0.165	0.168	0.171	0.174	0.175	0.176
Haikou	0.215	0.217	0.237	0.243	0.250	0.247	0.251	0.256	0.261	0.266	0.273	0.279	0.285	0.285	0.286
Chengdu	0.156	0.157	0.175	0.181	0.187	0.193	0.199	0.206	0.225	0.231	0.238	0.244	0.250	0.253	0.254
Guiyang	0.110	0.120	0.124	0.127	0.128	0.125	0.124	0.125	0.125	0.126	0.153	0.155	0.155	0.158	0.161
Kunming	0.137	0.137	0.140	0.140	0.140	0.142	0.142	0.143	0.163	0.167	0.171	0.175	0.179	0.181	0.183
Lhasa	0.171	0.174	0.186	0.190	0.188	0.190	0.193	0.196	0.196	0.197	0.199	0.200	0.237	0.238	0.238
Xinan	0.225	0.226	0.227	0.237	0.244	0.246	0.251	0.257	0.266	0.290	0.303	0.313	0.328	0.326	0.329
Lanzhou	0.130	0.130	0.141	0.141	0.141	0.144	0.145	0.146	0.147	0.148	0.149	0.151	0.175	0.176	0.177
Xining	0.393	0.396	0.393	0.395	0.396	0.400	0.402	0.405	0.407	0.410	0.412	0.415	0.416	0.417	0.417
Yinchuan	0.268	0.272	0.317	0.334	0.343	0.344	0.356	0.362	0.370	0.379	0.383	0.392	0.397	0.398	0.398
Urumchi	0.137	0.140	0.142	0.145	0.150	0.151	0.152	0.149	0.145	0.141	0.139	0.139	0.157	0.157	0.158

S4 Table. The scale of fiscal expenditure on public services of 27 provincial capital cities in China.

provincial capital cities	2008	2009	2010	2011	2012	2013	2014	2015	2016	2017	2018	2019	2020	2021	2022
Shijiazhuang	0.103	0.103	0.108	0.114	0.114	0.119	0.121	0.121	0.123	0.122	0.128	0.127	0.127	0.130	0.134
Taiyuan	0.116	0.102	0.098	0.101	0.101	0.105	0.105	0.123	0.124	0.128	0.127	0.130	0.127	0.125	0.122
Hohhot	0.092	0.086	0.078	0.086	0.080	0.079	0.080	0.085	0.093	0.089	0.074	0.082	0.083	0.080	0.072
Shenyang	0.189	0.177	0.162	0.164	0.168	0.170	0.180	0.180	0.180	0.175	0.181	0.182	0.179	0.176	0.168
Changchun	0.204	0.207	0.214	0.236	0.225	0.231	0.232	0.238	0.215	0.235	0.236	0.228	0.240	0.261	0.242
Harbin	0.195	0.186	0.201	0.199	0.203	0.211	0.216	0.205	0.207	0.207	0.206	0.220	0.213	0.194	0.195
Nanjing	0.125	0.115	0.110	0.107	0.110	0.109	0.109	0.108	0.118	0.127	0.131	0.132	0.128	0.125	0.123
Hangzhou	0.190	0.185	0.192	0.195	0.189	0.181	0.186	0.181	0.201	0.205	0.199	0.194	0.205	0.217	0.212
Hefei	0.126	0.115	0.123	0.144	0.144	0.145	0.150	0.147	0.156	0.156	0.153	0.152	0.156	0.161	0.165

Fuzhou	0.157	0.145	0.155	0.165	0.158	0.174	0.174	0.181	0.194	0.200	0.191	0.187	0.182	0.178	0.177
Nanchang	0.122	0.116	0.121	0.118	0.115	0.121	0.122	0.123	0.126	0.128	0.133	0.131	0.126	0.128	0.129
Jinan	0.082	0.080	0.081	0.079	0.079	0.078	0.080	0.080	0.085	0.090	0.101	0.111	0.115	0.110	0.101
Zhengzhou	0.127	0.121	0.125	0.133	0.140	0.146	0.152	0.163	0.177	0.184	0.191	0.188	0.166	0.166	0.137
Wuhan	0.291	0.241	0.396	0.398	0.236	0.257	0.238	0.218	0.237	0.253	0.266	0.281	0.285	0.279	0.258
Changsha	0.148	0.142	0.149	0.148	0.152	0.150	0.160	0.161	0.164	0.172	0.174	0.177	0.179	0.185	0.174
Guangzhou	0.189	0.182	0.180	0.176	0.182	0.165	0.157	0.135	0.145	0.145	0.159	0.166	0.169	0.166	0.163
Nanning	0.128	0.126	0.130	0.119	0.126	0.130	0.134	0.130	0.132	0.132	0.131	0.135	0.133	0.134	0.142
Haikou	0.131	0.137	0.137	0.128	0.125	0.131	0.137	0.138	0.146	0.137	0.141	0.143	0.156	0.139	0.159
Chengdu	0.172	0.167	0.183	0.184	0.180	0.187	0.197	0.196	0.199	0.202	0.189	0.194	0.193	0.200	0.204
Guiyang	0.135	0.124	0.125	0.123	0.127	0.128	0.127	0.128	0.123	0.126	0.124	0.121	0.118	0.122	0.124
Kunming	0.159	0.139	0.152	0.151	0.147	0.143	0.123	0.131	0.137	0.136	0.125	0.121	0.125	0.140	0.129
Lhasa	0.087	0.080	0.093	0.100	0.118	0.130	0.143	0.145	0.156	0.153	0.152	0.167	0.158	0.157	0.137
Xinan	0.159	0.150	0.167	0.169	0.180	0.199	0.207	0.210	0.215	0.216	0.217	0.218	0.227	0.243	0.232
Lanzhou	0.103	0.096	0.100	0.098	0.098	0.105	0.110	0.116	0.135	0.130	0.123	0.116	0.117	0.120	0.117
Xining	0.199	0.204	0.189	0.197	0.191	0.206	0.236	0.219	0.212	0.227	0.246	0.281	0.251	0.274	0.225
Yinchuan	0.194	0.168	0.215	0.209	0.216	0.239	0.264	0.253	0.264	0.249	0.256	0.241	0.225	0.204	0.225
Urumchi	0.094	0.099	0.094	0.104	0.109	0.115	0.122	0.117	0.101	0.099	0.132	0.117	0.097	0.078	0.067

S5 Table. The structure of fiscal expenditure on public services of 27 provincial capital cities in China.

provincial capital cities	2008	2009	2010	2011	2012	2013	2014	2015	2016	2017	2018	2019	2020	2021	2022
Urumchi	6.471	5.145	4.085	5.177	5.938	6.078	5.900	5.727	4.532	3.644	4.129	4.041	2.636	2.562	2.785
Lanzhou	8.548	5.990	5.933	4.797	4.537	4.342	5.338	5.721	6.091	5.814	6.033	6.612	5.027	5.674	3.879
Nanjing	5.434	5.139	4.765	4.909	4.633	4.771	4.308	3.949	4.216	3.860	4.060	3.744	2.497	3.154	2.883
Nanning	3.758	3.272	2.515	3.154	3.326	3.442	3.358	3.113	3.037	3.439	3.361	3.217	2.718	2.438	2.342
Nanchang	2.975	2.900	2.758	3.171	3.437	3.457	3.341	2.941	2.983	3.041	2.991	4.184	3.692	3.552	3.702

Hefei	5.025	4.774	6.875	4.729	5.053	5.110	5.103	4.662	4.726	4.669	4.725	5.117	4.471	5.225	3.899
Hohhot	5.034	5.109	4.361	4.470	4.257	3.682	4.313	4.350	4.796	4.078	3.424	4.745	3.618	2.908	2.523
Harbin	4.140	3.056	4.387	2.745	3.305	3.177	2.920	2.459	2.563	2.163	1.929	1.847	1.695	1.313	1.282
Taiyuan	2.801	2.402	3.191	3.602	3.885	3.817	3.651	3.566	3.370	3.112	3.444	3.949	3.545	3.018	2.819
Guangzhou	3.594	3.455	3.789	3.856	4.438	4.107	3.406	3.213	3.511	3.515	3.418	3.573	2.903	2.661	2.466
Chengdu	3.683	5.113	5.171	5.473	6.022	5.405	5.624	5.131	3.673	3.985	3.756	4.185	3.644	3.595	3.052
Lhasa	4.521	5.461	7.551	7.105	8.616	8.416	8.882	8.264	7.656	6.963	7.334	6.433	6.150	6.148	4.710
Kunming	3.194	3.320	3.623	3.636	4.262	4.302	3.606	3.563	3.633	3.946	3.326	3.325	3.261	3.047	2.667
Hangzhou	4.555	4.434	4.068	4.034	3.783	3.735	3.568	4.052	3.976	3.785	3.872	3.892	3.456	3.464	3.025
Wuhan	3.274	2.524	2.493	2.596	3.004	3.166	2.986	2.736	2.660	2.954	3.222	3.204	1.835	3.269	2.914
Shenyang	2.253	2.521	2.576	2.751	3.133	3.301	2.927	2.368	2.021	1.719	1.726	1.707	1.591	1.932	1.859
Jinan	3.454	3.373	3.743	3.562	3.451	3.440	3.321	3.239	3.117	3.193	3.671	3.461	3.301	2.775	2.427
Haikou	3.515	3.369	3.706	3.481	3.295	3.595	2.532	2.724	3.274	2.737	3.293	2.928	2.753	3.652	2.753
Shijiazhuang	3.992	4.287	4.500	4.721	4.364	4.386	4.158	3.929	3.492	3.115	2.852	3.250	2.863	2.858	3.154
Fuzhou	3.058	3.436	3.693	3.825	4.130	4.477	3.670	3.963	4.318	4.311	3.668	3.395	2.863	2.833	2.431
Xining	2.958	3.069	3.130	2.874	3.449	3.680	3.176	3.125	2.950	3.296	3.123	3.213	2.444	2.200	1.948
Xinan	2.200	2.788	2.846	3.263	3.571	3.865	3.807	3.633	3.614	3.287	3.515	3.924	2.874	3.002	2.659
Guiyang	4.191	4.365	4.312	4.645	5.130	5.063	4.710	4.687	4.648	4.131	4.632	5.162	4.029	3.744	3.207
Zhengzhou	4.099	4.087	3.899	4.242	4.487	4.395	4.707	5.130	5.694	6.356	6.363	5.362	4.676	4.636	3.700
Yinchuan	3.498	2.698	6.071	3.592	3.739	3.808	3.231	3.366	4.014	3.903	3.971	3.372	2.815	2.105	2.109
Changchun	2.854	2.777	3.150	3.155	3.530	3.425	3.554	2.997	2.831	3.434	3.118	3.046	2.837	2.793	1.781
Changsha	4.033	3.686	4.463	4.487	5.291	6.072	6.013	5.488	5.402	5.338	5.425	5.650	5.218	4.954	4.312

S6 Table. The urban size of 27 provincial capital cities in China.

provincial capital cities	2008	2009	2010	2011	2012	2013	2014	2015	2016	2017	2018	2019	2020	2021	2022
Urumchi	0.971	0.966	0.969	0.972	0.972	0.976	0.971	0.920	0.816	0.890	0.902	0.926	0.944	0.961	0.979
Lanzhou	0.609	0.613	0.763	0.773	0.783	0.797	0.803	0.810	0.810	0.810	0.810	0.810	0.831	0.836	0.841

Nanjing	0.770	0.772	0.779	0.797	0.802	0.805	0.809	0.814	0.820	0.823	0.825	0.832	0.868	0.869	0.870
Nanning	0.500	0.505	0.526	0.546	0.563	0.577	0.584	0.593	0.602	0.614	0.486	0.637	0.689	0.698	0.704
Nanchang	0.505	0.581	0.657	0.672	0.688	0.698	0.709	0.716	0.723	0.733	0.742	0.752	0.781	0.786	0.789
Hefei	0.432	0.641	0.685	0.646	0.664	0.678	0.691	0.704	0.721	0.738	0.750	0.763	0.823	0.840	0.846
Hohhot	0.603	0.610	0.625	0.648	0.672	0.695	0.708	0.723	0.741	0.756	0.767	0.780	0.792	0.797	0.798
Harbin	0.482	0.481	0.480	0.480	0.482	0.483	0.488	0.483	0.486	0.486	0.653	0.659	0.706	0.769	0.833
Taiyuan	0.820	0.822	0.825	0.834	0.838	0.841	0.843	0.844	0.846	0.847	0.849	0.853	0.891	0.892	0.893
Guangzhou	0.822	0.825	0.838	0.841	0.850	0.853	0.854	0.855	0.861	0.769	0.864	0.865	0.862	0.865	0.865
Chengdu	0.636	0.649	0.655	0.670	0.684	0.694	0.704	0.715	0.706	0.719	0.731	0.744	0.788	0.795	0.799
Lhasa	0.409	0.410	0.410	0.393	0.405	0.413	0.423	0.420	0.423	0.424	0.436	0.440	0.450	0.473	0.475
Kunming	0.604	0.610	0.636	0.660	0.671	0.681	0.691	0.701	0.711	0.721	0.729	0.736	0.797	0.805	0.813
Hangzhou	0.693	0.695	0.733	0.739	0.743	0.749	0.751	0.753	0.762	0.768	0.774	0.785	0.833	0.836	0.840
Wuhan	0.645	0.648	0.553	0.546	0.548	0.544	0.740	0.740	0.798	0.800	0.803	0.805	0.843	0.846	0.847
Shenyang	0.695	0.715	0.636	0.732	0.635	0.635	0.760	0.914	0.910	0.907	0.810	0.810	0.845	0.850	0.850
Jinan	0.714	0.713	0.714	0.715	0.716	0.660	0.664	0.680	0.695	0.705	0.721	0.712	0.735	0.742	0.743
Haikou	0.604	0.604	0.603	0.601	0.755	0.761	0.766	0.772	0.778	0.782	0.786	0.788	0.818	0.826	0.835
Shijiazhuang	0.412	0.492	0.420	0.440	0.451	0.463	0.476	0.583	0.600	0.616	0.632	0.644	0.702	0.711	0.714
Fuzhou	0.575	0.590	0.620	0.633	0.648	0.659	0.669	0.677	0.685	0.695	0.703	0.705	0.725	0.730	0.733
Xining	0.610	0.614	0.637	0.654	0.677	0.678	0.686	0.689	0.700	0.711	0.721	0.729	0.786	0.793	0.799
Xinan	0.732	0.744	0.690	0.701	0.715	0.721	0.726	0.730	0.734	0.734	0.740	0.746	0.792	0.795	0.796
Guiyang	0.695	0.705	0.629	0.693	0.705	0.721	0.732	0.733	0.742	0.748	0.754	0.761	0.801	0.803	0.804
Zhengzhou	0.623	0.634	0.636	0.648	0.663	0.671	0.683	0.697	0.710	0.722	0.734	0.746	0.784	0.791	0.794
Yinchuan	0.699	0.711	0.725	0.743	0.751	0.748	0.755	0.758	0.757	0.771	0.776	0.791	0.802	0.814	0.827
Changchun	0.480	0.479	0.478	0.479	0.480	0.483	0.485	0.579	0.581	0.585	0.588	0.591	0.659	0.668	0.677
Changsha	0.613	0.626	0.677	0.685	0.694	0.706	0.723	0.744	0.760	0.776	0.791	0.796	0.826	0.832	0.833

S7 Table. The basic public service expenditure of the other city of 27 provincial capital cities in China.

provincial capital cities	2008	2009	2010	2011	2012	2013	2014	2015	2016	2017	2018	2019	2020	2021	2022
Shijiazhuang	0.180	0.196	0.199	0.199	0.213	0.228	0.232	0.265	0.272	0.250	0.242	0.222	0.205	0.185	0.176
Taiyuan	0.142	0.152	0.150	0.149	0.162	0.196	0.195	0.216	0.202	0.190	0.209	0.217	0.229	0.168	0.175
Hohhot	0.147	0.171	0.187	0.221	0.210	0.209	0.207	0.214	0.195	0.151	0.173	0.174	0.189	0.147	0.043
Shenyang	0.139	0.145	0.119	0.178	0.198	0.221	0.191	0.169	0.154	0.152	0.154	0.145	0.143	0.125	0.079
Changchun	0.104	0.110	0.119	0.114	0.117	0.125	0.138	0.149	0.155	0.182	0.186	0.297	0.307	0.211	0.262
Harbin	0.058	0.068	0.057	0.058	0.059	0.057	0.058	0.116	0.127	0.132	0.120	0.124	0.150	0.129	0.123
Nanjing	0.189	0.201	0.196	0.196	0.196	0.210	0.209	0.246	0.246	0.246	0.256	0.214	0.209	0.195	0.170
Hangzhou	0.189	0.201	0.196	0.196	0.196	0.210	0.209	0.246	0.246	0.246	0.256	0.214	0.209	0.195	0.170
Hefei	0.112	0.120	0.109	0.146	0.164	0.154	0.151	0.166	0.156	0.161	0.139	0.140	0.131	0.118	0.129
Fuzhou	0.060	0.061	0.064	0.070	0.075	0.081	0.083	0.088	0.090	0.085	0.075	0.066	0.071	0.059	0.067
Nanchang	0.167	0.200	0.192	0.205	0.238	0.251	0.253	0.266	0.266	0.277	0.271	0.291	0.267	0.230	0.227
Jinan	0.089	0.096	0.099	0.105	0.112	0.135	0.132	0.141	0.146	0.138	0.142	0.134	0.128	0.121	0.085
Zhengzhou	0.089	0.102	0.136	0.110	0.152	0.162	0.167	0.163	0.136	0.128	0.129	0.129	0.136	0.119	0.111
Wuhan	0.101	0.123	0.126	0.111	0.118	0.128	0.141	0.159	0.143	0.172	0.152	0.152	0.148	0.114	0.108
Changsha	0.102	0.103	0.107	0.108	0.107	0.115	0.118	0.130	0.139	0.157	0.156	0.141	0.110	0.122	0.121
Guangzhou	0.112	0.118	0.126	0.133	0.116	0.111	0.129	0.191	0.204	0.197	0.169	0.169	0.151	0.149	0.154
Nanning	0.108	0.126	0.112	0.118	0.125	0.125	0.124	0.137	0.140	0.139	0.141	0.160	0.154	0.139	0.117
Haikou	0.181	0.220	0.224	0.250	0.255	0.242	0.261	0.268	0.255	0.238	0.287	0.313	0.287	0.242	0.270
Chengdu	0.172	0.198	0.219	0.253	0.263	0.235	0.226	0.236	0.222	0.216	0.210	0.205	0.196	0.172	0.168
Guiyang	0.200	0.204	0.220	0.232	0.242	0.235	0.218	0.218	0.218	0.232	0.214	0.215	0.203	0.186	0.163
Kunming	0.148	0.161	0.181	0.184	0.201	0.188	0.216	0.220	0.225	0.228	0.203	0.205	0.180	0.148	0.133
Lhasa	0.638	0.652	0.427	0.545	0.681	0.874	0.856	1.224	1.150	1.136	1.206	1.399	1.222	0.982	0.761
Xinan	0.108	0.136	0.141	0.143	0.153	0.154	0.145	0.189	0.173	0.169	0.170	0.179	0.170	0.142	0.164
Lanzhou	0.264	0.301	0.328	0.157	0.155	0.151	0.288	0.357	0.221	0.244	0.230	0.255	0.236	0.203	0.218
Xining	0.331	0.384	0.404	0.487	0.465	0.408	0.493	0.542	0.486	0.454	0.478	0.478	0.456	0.362	0.398
Yinchuan	0.200	0.242	0.318	0.264	0.289	0.397	0.375	0.387	0.399	0.394	0.383	0.378	0.374	0.297	0.284

Urumchi	0.070	0.271	0.299	0.322	0.341	0.324	0.301	0.312	0.367	0.402	0.363	0.359	0.343	0.300	0.353

S8 Table. The local government scale of 27 provincial capital cities in China.

provincial capital cities	2008	2009	2010	2011	2012	2013	2014	2015	2016	2017	2018	2019	2020	2021	2022
Urumchi	0.099	0.104	0.113	0.136	0.126	0.137	0.138	0.140	0.150	0.147	0.148	0.152	0.118	0.102	0.081
Lanzhou	0.059	0.061	0.064	0.062	0.064	0.069	0.077	0.088	0.098	0.096	0.095	0.082	0.086	0.086	0.066
Nanjing	0.100	0.101	0.104	0.102	0.100	0.101	0.101	0.102	0.106	0.107	0.113	0.112	0.111	0.106	0.092
Nanning	0.154	0.085	0.093	0.091	0.100	0.098	0.096	0.094	0.092	0.087	0.086	0.082	0.079	0.077	0.075
Nanchang	0.062	0.065	0.068	0.071	0.083	0.091	0.098	0.103	0.097	0.092	0.090	0.086	0.084	0.072	0.064
Hefei	0.133	0.086	0.096	0.093	0.093	0.093	0.095	0.098	0.094	0.089	0.083	0.080	0.076	0.074	0.076
Hohhot	0.088	0.099	0.068	0.107	0.115	0.105	0.113	0.118	0.121	0.083	0.079	0.073	0.077	0.072	0.069
Harbin	0.084	0.088	0.065	0.103	0.111	0.114	0.112	0.100	0.086	0.078	0.077	0.072	0.066	0.068	0.048
Taiyuan	0.079	0.079	0.078	0.085	0.095	0.104	0.105	0.103	0.103	0.095	0.100	0.097	0.091	0.083	0.078
Guangzhou	0.074	0.077	0.082	0.080	0.084	0.076	0.077	0.078	0.075	0.077	0.078	0.072	0.069	0.067	0.064
Chengdu	0.084	0.082	0.096	0.093	0.091	0.095	0.099	0.109	0.099	0.092	0.091	0.087	0.085	0.085	0.083
Lhasa	0.058	0.101	0.084	0.119	0.155	0.165	0.239	0.236	0.159	0.178	0.194	0.189	0.158	0.144	0.096
Kunming	0.107	0.108	0.117	0.124	0.124	0.128	0.122	0.116	0.111	0.103	0.099	0.097	0.097	0.095	0.067
Hangzhou	0.094	0.102	0.111	0.112	0.110	0.113	0.108	0.118	0.120	0.119	0.128	0.128	0.129	0.131	0.131
Wuhan	0.068	0.067	0.071	0.102	0.107	0.198	0.110	0.118	0.115	0.107	0.102	0.096	0.079	0.089	0.080
Shenyang	0.099	0.099	0.093	0.155	0.163	0.168	0.155	0.116	0.117	0.118	0.115	0.113	0.112	0.107	0.093
Jinan	0.062	0.063	0.068	0.074	0.079	0.092	0.094	0.101	0.097	0.095	0.096	0.093	0.089	0.088	0.083
Haikou	0.071	0.080	0.080	0.085	0.085	0.088	0.092	0.098	0.089	0.088	0.111	0.110	0.104	0.101	0.096
Shijiazhuang	0.040	0.042	0.059	0.066	0.076	0.081	0.085	0.088	0.088	0.092	0.097	0.098	0.107	0.105	0.097
Fuzhou	0.071	0.073	0.081	0.084	0.088	0.093	0.095	0.097	0.092	0.085	0.080	0.071	0.067	0.066	0.057
Xining	0.052	0.056	0.055	0.060	0.065	0.069	0.078	0.084	0.060	0.062	0.072	0.077	0.098	0.099	0.080
Xinan	0.063	0.067	0.076	0.084	0.091	0.101	0.105	0.110	0.100	0.088	0.081	0.075	0.072	0.080	0.073
Guiyang	0.110	0.111	0.122	0.137	0.148	0.139	0.145	0.250	0.116	0.107	0.110	0.103	0.092	0.086	0.082

Zhengzhou	0.086	0.091	0.096	0.101	0.109	0.117	0.123	0.128	0.124	0.114	0.108	0.106	0.106	0.096	0.087
Yinchuan	0.063	0.070	0.084	0.101	0.106	0.114	0.122	0.130	0.123	0.110	0.098	0.082	0.080	0.076	0.067
Changchun	0.055	0.058	0.062	0.081	0.085	0.087	0.083	0.081	0.083	0.083	0.085	0.071	0.067	0.087	0.068
Changsha	0.065	0.068	0.069	0.078	0.079	0.078	0.084	0.085	0.081	0.080	0.085	0.082	0.091	0.090	0.086

S9 Table. The industry structures of 27 provincial capital cities in China.

provincial capital cities	2008	2009	2010	2011	2012	2013	2014	2015	2016	2017	2018	2019	2020	2021	2022
Urumchi	0.770	0.784	0.859	0.872	0.762	0.717	0.641	0.473	0.408	0.438	0.448	0.365	0.378	0.396	0.415
Lanzhou	0.925	0.926	0.953	0.969	0.929	0.813	0.764	0.673	0.572	0.583	0.565	0.509	0.492	0.536	0.541
Nanjing	0.951	0.890	0.916	0.857	0.825	0.792	0.745	0.703	0.671	0.637	0.603	0.579	0.560	0.582	0.577
Nanning	0.522	0.472	0.501	0.534	0.512	0.484	0.490	0.465	0.419	0.386	0.364	0.354	0.349	0.362	0.344
Nanchang	1.447	1.436	1.500	1.620	1.455	1.393	1.358	1.322	1.236	1.243	1.098	0.972	0.945	1.008	1.004
Hefei	1.152	1.244	1.310	1.404	1.412	1.403	1.416	1.348	1.133	1.105	0.918	0.599	0.584	0.605	0.607
Hohhot	0.494	0.510	0.523	0.536	0.552	0.586	0.575	0.497	0.496	0.475	0.465	0.444	0.456	0.558	0.574
Harbin	0.769	0.760	0.741	0.766	0.682	0.584	0.609	0.579	0.540	0.473	0.413	0.317	0.334	0.356	0.364
Taiyuan	1.050	0.806	0.842	0.865	0.835	0.796	0.684	0.608	0.578	0.614	0.599	0.615	0.575	0.713	0.807
Guangzhou	0.660	0.612	0.611	0.599	0.548	0.525	0.513	0.479	0.441	0.394	0.380	0.381	0.363	0.382	0.384
Chengdu	1.001	0.896	0.891	0.904	0.936	0.914	0.869	0.828	0.801	0.812	0.785	0.470	0.465	0.463	0.463
Lhasa	0.427	0.442	0.460	0.509	0.523	0.518	0.548	0.551	0.572	0.594	0.655	0.653	0.795	0.633	0.677
Kunming	0.974	0.948	0.925	0.956	0.936	0.903	0.872	0.724	0.681	0.671	0.692	0.504	0.487	0.497	0.503
Hangzhou	1.080	0.984	0.983	0.962	0.925	0.829	0.761	0.668	0.588	0.558	0.530	0.479	0.440	0.447	0.440
Wuhan	0.920	0.920	0.900	0.985	1.010	1.018	0.970	0.895	0.830	0.821	0.787	0.608	0.576	0.561	0.575
Shenyang	1.110	1.137	1.134	1.161	1.165	1.191	1.096	1.017	0.698	0.678	0.650	0.544	0.526	0.590	0.645
Jinan	1.155	0.850	0.796	0.782	0.739	0.710	0.703	0.661	0.615	0.595	0.595	0.560	0.565	0.562	0.563
Haikou	0.362	0.320	0.319	0.309	0.295	0.280	0.266	0.248	0.234	0.229	0.222	0.204	0.212	0.217	0.249
Shijiazhuang	1.290	1.246	1.201	1.242	1.240	1.171	1.067	0.984	0.963	0.950	0.677	0.519	0.473	0.543	0.555
Fuzhou	0.849	0.876	0.960	0.948	0.967	0.981	0.972	0.901	0.850	0.786	0.774	0.706	0.684	0.671	0.668

Xining	1.295	1.072	0.429	0.583	0.601	0.499	0.450	0.398	0.388	0.462	0.466	0.454	0.467	0.533	0.642
Xinan	0.899	0.787	0.833	0.851	0.831	0.831	0.722	0.632	0.574	0.565	0.566	0.539	0.522	0.528	0.574
Guiyang	0.995	0.893	0.752	0.800	0.788	0.735	0.691	0.671	0.676	0.682	0.633	0.628	0.602	0.593	0.584
Zhengzhou	1.328	1.259	1.182	1.456	1.377	1.343	1.110	0.957	0.836	0.774	0.701	0.658	0.665	0.660	0.683
Yinchuan	0.862	0.889	0.935	1.026	1.034	0.992	0.978	0.914	0.878	0.902	0.833	0.826	0.778	0.933	1.067
Changchun	1.271	1.221	1.268	1.292	1.240	1.321	1.314	1.147	1.092	1.045	1.044	0.815	0.824	0.818	0.770
Changsha	1.243	1.133	1.277	1.417	1.417	1.354	1.296	1.214	1.017	0.969	0.774	0.655	0.679	0.692	0.705

S10 Table. The foreign trade level of 27 provincial capital cities in China.

provincial capital cities	2008	2009	2010	2011	2012	2013	2014	2015	2016	2017	2018	2019	2020	2021	2022
Urumchi	0.36	0.23	0.31	0.35	0.33	0.22	0.21	0.14	0.13	0.17	0.17	0.17	0.14	0.10	0.13
Lanzhou	0.06	0.04	0.06	0.09	0.13	0.14	0.14	0.17	0.13	0.05	0.05	0.04	0.04	0.04	0.05
Nanjing	0.73	0.54	0.62	0.59	0.48	0.42	0.39	0.33	0.31	0.35	0.33	0.34	0.36	0.39	0.37
Nanning	0.10	0.13	0.09	0.08	0.11	0.10	0.10	0.12	0.12	0.16	0.18	0.17	0.21	0.24	0.29
Nanchang	0.14	0.13	0.17	0.19	0.18	0.19	0.21	0.19	0.15	0.15	0.15	0.19	0.20	0.19	0.19
Hefei	0.32	0.21	0.25	0.22	0.27	0.24	0.24	0.22	0.19	0.23	0.24	0.24	0.26	0.29	0.30
Hohhot	0.07	0.04	0.05	0.09	0.07	0.06	0.07	0.06	0.04	0.04	0.04	0.04	0.05	0.05	0.06
Harbin	0.13	0.11	0.08	0.11	0.11	0.11	0.11	0.07	0.06	0.05	0.04	0.05	0.05	0.06	0.07
Taiyuan	0.44	0.27	0.30	0.27	0.24	0.24	0.27	0.25	0.32	0.28	0.29	0.28	0.29	0.36	0.26
Guangzhou	0.68	0.57	0.66	0.62	0.56	0.49	0.50	0.48	0.46	0.49	0.47	0.42	0.38	0.38	0.38
Chengdu	0.25	0.26	0.30	0.33	0.35	0.33	0.33	0.23	0.23	0.28	0.32	0.34	0.40	0.41	0.40
Lhasa	0.02	0.01	0.31	0.37	0.80	0.64	0.37	0.11	0.09	0.09	0.07	0.07	0.02	0.05	0.06
Kunming	0.31	0.21	0.32	0.30	0.30	0.31	0.28	0.18	0.09	0.10	0.14	0.14	0.17	0.24	0.26
Hangzhou	0.69	0.54	0.59	0.59	0.50	0.48	0.44	0.39	0.38	0.39	0.37	0.36	0.37	0.40	0.40
Wuhan	0.24	0.16	0.22	0.23	0.17	0.15	0.16	0.17	0.14	0.15	0.14	0.15	0.17	0.19	0.19
Shenyang	0.17	0.14	0.11	0.17	0.18	0.19	0.19	0.17	0.14	0.16	0.16	0.17	0.16	0.20	0.18
Jinan	0.18	0.12	0.13	0.15	0.12	0.11	0.11	0.09	0.11	0.11	0.11	0.12	0.14	0.17	0.18

Haikou	0.57	0.54	0.42	0.36	0.31	0.32	0.19	0.24	0.20	0.15	0.22	0.20	0.21	0.23	0.28
Shijiazhuang	0.18	0.13	0.27	0.27	0.23	0.22	0.22	0.18	0.16	0.17	0.17	0.20	0.23	0.23	0.17
Fuzhou	0.59	0.46	0.53	0.57	0.45	0.39	0.40	0.36	0.32	0.31	0.29	0.27	0.25	0.29	0.30
Xining	0.10	0.06	0.07	0.07	0.07	0.08	0.09	0.10	0.07	0.03	0.02	0.02	0.01	0.01	0.02
Xinan	0.21	0.18	0.22	0.21	0.19	0.22	0.28	0.30	0.29	0.34	0.39	0.35	0.35	0.41	0.39
Guiyang	0.19	0.13	0.14	0.18	0.20	0.20	0.21	0.20	0.08	0.06	0.06	0.07	0.10	0.11	0.12
Zhengzhou	0.10	0.07	0.09	0.21	0.41	0.43	0.42	0.48	0.45	0.43	0.38	0.36	0.42	0.46	0.47
Yinchuan	0.15	0.07	0.09	0.08	0.08	0.13	0.22	0.23	0.12	0.17	0.09	0.08	0.03	0.06	0.06
Changchun	0.28	0.24	0.31	0.32	0.31	0.29	0.27	0.18	0.19	0.18	0.19	0.17	0.16	0.17	0.16
Changsha	0.11	0.08	0.09	0.09	0.09	0.09	0.10	0.09	0.08	0.09	0.12	0.17	0.19	0.21	0.24

S11 Table. The population intension of 27 provincial capital cities in China.

provincial capital cities	2008	2009	2010	2011	2012	2013	2014	2015	2016	2017	2018	2019	2020	2021	2022
Shijiazhuang	6.10	6.23	6.42	6.49	6.55	6.63	6.70	6.75	6.81	6.87	6.91	6.96	7.09	7.71	7.72
Taiyuan	4.97	5.01	6.02	6.20	6.36	6.50	6.60	6.75	6.94	7.13	7.32	7.49	7.61	7.71	7.78
Hohhot	1.61	1.64	1.67	1.71	1.76	1.80	1.84	1.87	1.91	1.94	1.96	1.98	2.01	2.03	2.07
Shenyang	5.54	6.11	6.30	6.36	6.40	6.42	6.44	6.45	6.69	6.77	6.88	6.96	7.06	7.09	7.11
Changchun	3.75	3.74	3.73	3.73	3.72	3.72	3.70	3.70	3.69	3.68	3.67	3.66	4.40	3.70	3.69
Harbin	1.96	1.98	2.00	1.99	1.98	1.97	1.96	1.94	2.06	2.06	2.05	2.03	1.89	1.86	1.86
Nanjing	11.53	11.72	12.17	12.31	12.39	12.43	12.47	12.50	12.56	12.65	12.81	12.90	14.15	14.31	14.41
Hangzhou	4.94	4.88	5.25	5.27	5.30	5.33	6.11	6.25	6.39	6.58	6.78	7.00	7.21	7.24	7.34
Hefei	7.11	7.24	10.58	6.58	6.62	6.65	6.72	7.26	7.44	7.63	7.80	8.01	8.19	8.27	8.42
Fuzhou	5.71	5.74	5.95	6.14	6.24	6.35	6.48	6.52	6.58	6.73	6.83	6.89	6.95	6.71	7.06
Nanchang	6.24	6.28	6.83	6.98	7.13	7.25	7.41	7.56	7.76	8.00	8.13	8.30	8.45	8.95	9.09
Jinan	8.10	8.17	8.34	8.42	8.50	8.56	8.84	8.94	9.05	9.15	9.33	11.14	11.51	9.11	9.19
Zhengzhou	9.99	10.10	11.63	12.21	12.73	13.26	13.83	12.85	15.03	15.63	16.18	16.59	16.95	16.84	16.95
Wuhan	10.56	10.71	11.52	11.80	11.91	12.03	12.06	12.38	12.56	12.71	12.93	13.08	14.53	15.93	16.03
Changsha	5.57	5.62	5.96	6.27	6.48	6.66	6.88	7.01	7.27	7.64	7.85	8.15	8.51	8.67	8.82
Guangzhou	15.00	15.97	17.10	18.11	19.04	19.80	20.57	21.45	22.58	23.49	24.19	24.63	25.21	25.30	25.20
Nanning	3.15	3.17	3.02	3.12	3.20	3.29	3.37	3.46	3.56	3.67	3.77	3.86	3.96	4.00	4.02

Haikou	7.96	8.15	8.92	9.26	9.63	9.86	10.29	10.57	10.93	11.33	11.73	12.14	12.64	12.66	12.80
Chengdu	10.26	10.61	11.59	12.02	12.47	12.91	13.36	13.90	15.33	15.83	16.35	16.84	17.28	14.78	14.84
Guiyang	4.90	5.27	5.39	5.46	5.54	5.63	5.66	5.75	5.84	5.97	7.25	7.39	7.45	7.59	7.73
Kunming	2.97	2.99	3.06	3.09	3.11	3.13	3.15	3.18	3.62	3.72	3.82	3.93	4.03	4.05	4.09
Lhasa	0.17	0.17	0.19	0.20	0.20	0.20	0.21	0.22	0.23	0.23	0.24	0.24	0.29	0.29	0.29
Xinan	8.29	8.34	8.38	8.78	9.04	9.26	9.51	9.79	10.20	11.23	11.80	12.23	12.84	12.74	12.86
Lanzhou	2.53	2.54	2.77	2.77	2.77	2.78	2.80	2.82	2.83	2.85	2.87	2.90	3.34	3.32	3.35
Xining	2.83	2.87	2.88	2.93	2.97	2.99	3.03	3.05	3.10	3.14	3.16	3.20	3.23	3.23	3.24
Yinchuan	1.73	1.78	2.10	2.36	2.46	2.54	2.67	2.74	2.85	2.96	3.01	3.11	3.17	3.19	3.21
Urumchi	2.06	2.19	2.26	2.33	2.43	2.51	2.56	2.57	2.55	2.54	2.54	2.58	2.94	2.95	2.96

Section 5. The structure on fiscal expenditure.

S12 Table. The specific division of the structure on fiscal expenditure.

Group	Classification
Purchase Spending (a)	General Public Services
	Public Safety
	Urban and Rural Community Management
Purchase Spending (b)	Education
	Culture, Sports and Media
	Transportation
	Agriculture, Forestry and Water Management
	Environmental Protection
	Science and Technology
Transfer Spending	Social Security and Employment
	Medical and Healthcare

Section 6. Method.

GMM.

The general form of the equation of 2S-GMM is as follows:

$$y_{i,t} = \gamma + \alpha_1 y_{i,t-1} + \alpha_2 y_{i,t-2} + \cdots + \alpha_p y_{i,t-p} + \boldsymbol{\beta x}_{i,t} + \boldsymbol{\delta_i} Control\ variables_{i,t} + u_i + \varepsilon_i$$

In this equation, $\mathrm{y}_{i,t}$ represented the explained variables, $\mathrm{y}_{i,t-p}$represented P-order lag term of the explained variables, $x_{i,t}$ represented the explanatory variables, z_i represented the time invariant individual characteristics, $u_i + \varepsilon_i$ represented the compound random disturbance term, γ represented the constant term, α_p represented the coefficient of the lag term of explained variables, β represented the coefficient of explanatory variables, δ_i represented the coefficient of individual characteristics.

PTR.

In linear regression, all observed samples have the same regression function, so if all observed samples are discrete, how will the regression function differ? In regression analysis, we are often concerned about whether the coefficient estimate is stable, that is, if the whole sample is divided into several subsamples for regression, can we still get roughly the same estimated coefficient? If the variable of the sample is not a discrete variable, but a continuous variable, it is necessary to give a criterion, that is, a "threshold value". The threshold effect model proposed by Hansen (1999) is designed for non-dynamic panels with individual fixed effects [S6]. The threshold value can be transformed by fixed effects and the regression slope can be obtained by least squares estimation. Inference is made using a non-standard asymptotic theory and allows for the

construction of confidence intervals for hypothesis testing. Caner and Hansen (2004) have taken a more indirect approach to the problem [S7]. They studied a panel threshold model with endogenous explanatory variables and an exogenous threshold variable. As long as the endogenous variables are treated with a simplified form, and then 2SLS or GMM are used to estimate the parameters, the requirement for endogenous explanatory variables is relaxed. However, the requirement for threshold variables is still that exogenous variables must be strong. This paper is used to characterize the dynamic threshold effect model.

Section 7 Comparative experiment of empirical research

In the empirical research part, comparative experiments are needed to select which regression method to use for panel data. Through the tests of F, Hausman, Abond and Sargan, GMM was chosen to achieve the empirical analysis. Meanwhile, by comparing the estimated value of the L.AE for OLS, FE, D-GMM and S-GMM, system GMM was chosen to achieve the empirical analysis, eventually.

S13 Table. The results of comparative experiments.

Variables	OLS	FE	RE	D-GMM	S-GMM
L.AE	0.952***	0.708***	0.952***	0.061***	0.734***
	(0.014)	(0.039)	(0.014)	(0.009)	(0.020)
FEscale	0.083***	0.085	0.083***	0.010	0.311***
	(0.025)	(0.052)	(0.025)	(0.039)	(0.048)
LGS	-0.029	-0.061	-0.029	-0.073*	-0.129***
	(0.034)	(0.048)	(0.034)	(0.043)	(0.043)
IS	-0.002	-0.019***	-0.002	-0.043***	0.012***
	(0.003)	(0.006)	(0.003)	(0.003)	(0.002)
Open	-0.002	-0.008	-0.002	-0.043***	-0.030***
	(0.008)	(0.011)	(0.008)	(0.007)	(0.004)
CS	-0.001	-0.001	-0.000	0.003***	0.003***
	(0.000)	(0.001)	(0.000)	(0.001)	(0.000)
Constant	0.008	0.092***	0.008	0.258***	0.011***
	(0.005)	(0.017)	(0.005)	(0.010)	(0.003)
**F Test**	1944.85	87.91	11669.12	/	/
	(0.0000	(0.0000)	(0.0000)	/	/
**R^2**	0.9687	0.6049	0.5914	/	/
**Hausman Test**	/	42.22		/	/

**AR (1) Test**	/	/	/	0.1441	0.0214
**AR (2) Test**	/	/	/	0.9681	0.4811
**Sargan Test**	/	/	/	0.5559	0.9880

*** p<0.01, ** p<0.05, * p<0.1. t-value in parentheses, Arellano-Bond tests for AR (1) and AR (2), Sargan tests for Sargan, and Wald tests for Wald.

Section 8: Threshold tests.

PTR Model 1- PTR Model 12.

S14 Table. The tests of threshold effect of the general equilibrium for PTR Model 1- Model 12, corresponding to the Fig. 3 of the manuscript.

Explained Variable	Explaining Variable	Threshold Variable	The type of threshold effect	F	P	Critical Value		
						10%	5%	1%
AE	*FEscale*	*Urban*	*Single*	86.46	0.000	20.556	26.426	36.782
			Double	27.41	0.023	17.414	21.975	31.403
			Triple	12.91	0.580	33.184	38.772	51.797
		FEother	*Single*	46.83	0.010	20.074	25.031	35.834
			Double	22.88	0.013	14.995	18.750	23.229
			Triple	13.05	0.375	22.604	28.597	37.013
	FEstructure	*Urban*	*Single*	26.79	0.068	23.917	28.949	39.708
			Double	19.17	0.103	19.250	25.399	34.641
			Triple	14.98	0.585	40.422	50.120	79.163
		FEother	*Single*	18.88	0.070	17.073	20.301	27.570
			Double	8.08	0.400	14.580	17.350	23.711
			Triple	9.57	0.175	12.471	15.454	23.072
IAE	*FEscale*	*Urban*	*Single*	90.72	0.000	14.250	15.562	24.057
			Double	16.68	0.048	13.600	15.875	27.769
			Triple	4.98	0.800	19.046	24.228	36.828
		FEother	*Single*	30.44	0.028	21.675	27.164	43.334
			Double	22.41	0.043	16.749	21.484	27.236
			Triple	7.80	0.613	22.825	28.850	45.766
	FEstructure	*Urban*	*Single*	48.75	0.000	18.808	21.655	27.749
			Double	13.44	0.150	15.834	18.624	28.635
			Triple	3.80	0.740	13.957	16.697	26.070
		FEother	*Single*	18.02	0.150	20.156	24.443	33.854
			Double	9.35	0.273	13.745	18.951	26.539
			Triple	3.74	0.750	11.736	13.691	19.400
PAE	*FEscale*	*Urban*	*Single*	66.61	0.015	39.599	51.029	70.928
			Double	50.22	0.015	32.726	40.095	52.911
			Triple	20.02	0.615	55.506	65.741	85.309

		FEother	*Single*	49.83	0.003	19.656	24.265	33.643
			Double	25.75	0.075	22.055	28.703	38.661
			Triple	7.15	0.703	25.897	31.009	42.961
	FEstructure	*Urban*	*Single*	38.34	0.088	36.939	49.783	69.624
			Double	26.14	0.173	33.053	38.600	50.099
			Triple	8.60	0.690	27.067	33.963	43.727
		FEother	*Single*	10.70	0.393	21.704	26.909	35.020
			Double	5.29	0.705	15.961	19.679	31.950
			Triple	0.80	1.000	10.506	12.385	16.861

S15 Table. The results of threshold effect of the general equilibrium for PTR Model 1-Model 4.

Variable	M1	M2	M3	M4
$\boldsymbol{LGS}$	-0.161***	-0.316***	-0.110*	-0.180***
	(0.054)	(0.058)	(0.061)	(0.062)
$\boldsymbol{IS}$	-0.027***	-0.043***	-0.047***	-0.057***
	(0.007)	(0.007)	(0.008)	(0.007)
$\boldsymbol{Open}$	-0.004	0.008	-0.003	-0.010
	(0.012)	(0.013)	(0.014)	(0.015)
$\boldsymbol{PI}$	-0.001	-0.001	-0.001	-0.001
	(0.001)	(0.001)	(0.001)	(0.001)
$\boldsymbol{FEscale(Urban \leq \gamma_1)}$	-0.006			
	(0.071)			
$\boldsymbol{FEscale(\gamma_1 < Urban \leq \gamma_2)}$	0.173***			
	(0.062)			
$\boldsymbol{FEscale(Urban > \gamma_2)}$	0.349***			
	(0.064)			
$\boldsymbol{FEscale(FEother \leq \gamma_1)}$		0.114*		
		(0.066)		
$\boldsymbol{FEscale(\gamma_1 < FEother \leq \gamma_2)}$		0.217***		
		(0.065)		
$\boldsymbol{FEscale(FEother > \gamma_2)}$		0.382***		
		(0.067)		
$\boldsymbol{FEstructure\ (Urban \leq \gamma_1)}$			-0.006***	
			(0.002)	
$\boldsymbol{FEstructure\ (Urban > \gamma_1)}$			0.002	
			(0.001)	
$\boldsymbol{FEstructure(FEother \leq \gamma_1)}$				-0.003
				(0.002)

FEstructure $(\boldsymbol{FEother} > \boldsymbol{\gamma_1})$				0.001
				(0.002)
Constant	0.261***	0.297***	0.315***	0.341***
	(0.015)	(0.015)	(0.014)	(0.014)
F	33.12	26.74	19.70	18.15
	(0.0000)	(0.0000)	(0.0000)	(0.0000)
R2	0.3846	0.3354	0.2411	0.2264

Note: *** p<0.01, ** p<0.05, * p<0.1. Standard errors in parentheses.

S16 Table. The results of threshold effect of the general equilibrium for PTR Model 5-Model 8.

Variable	M5	M6	M7	M8
LGS	0.262***	0.118	0.308***	No threshold value
	(0.073)	(0.079)	(0.079)	
IS	0.002	-0.009	-0.001	
	(0.010)	(0.010)	(0.010)	
Open	0.011	0.003	0.033*	
	(0.017)	(0.018)	(0.020)	
PI	0.003**	-0.001	0.002	
	(0.001)	(0.001)	(0.001)	
FEscale $(\boldsymbol{Urban} \le \boldsymbol{\gamma_1})$	-0.257***			
	(0.097)			
FEscale $(\boldsymbol{\gamma_1} < \boldsymbol{Urban} \le \boldsymbol{\gamma_2})$	0.215**			
	(0.084)			
FEscale $(\boldsymbol{Urban} > \boldsymbol{\gamma_2})$	0.117			
	(0.086)			
FEscale $(\boldsymbol{FEother} \le \boldsymbol{\gamma_1})$		-0.147		
		(0.107)		
FEscale $(\boldsymbol{\gamma_1} < \boldsymbol{FEother} \le \boldsymbol{\gamma_2})$		0.165*		
		(0.089)		
FEscale $(\boldsymbol{FEother} > \boldsymbol{\gamma_2})$		0.302***		
		(0.091)		
FEstructure $(\boldsymbol{Urban} \le \boldsymbol{\gamma_1})$			-0.016***	
			(0.003)	
FEstructure $(\boldsymbol{Urban} > \boldsymbol{\gamma_1})$			-8.24e-05	
			(0.002)	
Constant	0.240***	0.266***	0.259***	
	(0.021)	(0.021)	(0.019)	

F	18.23	10.84	11.51	
	(0.0000)	(0.0000)	(0.0000)	
R2	0.2559	0.1698	0.1565	

Note: *** p<0.01, ** p<0.05, * p<0.1. Standard errors in parentheses.

S17 Table. The results of threshold effect of the general equilibrium for PTR Model 9-Model 12.

Variable	M9	M10	M11	M12
LGS	-0.096***	-0.191***	0.026	No threshold value
	(0.033)	(0.036)	(0.039)	
IS	-0.005	-0.025***	-0.016***	
	(0.004)	(0.004)	(0.005)	
Open	-0.009	-0.001	0.005	
	(0.008)	(0.008)	(0.009)	
PI	0.007***	0.007***	0.008***	
	(0.001)	(0.001)	(0.001)	
$FEscale(Urban \le \gamma_1)$	0.257***			
	(0.038)			
$FEscale(\gamma_1 < Urban \le \gamma_2)$	0.361***			
	(0.039)			
$FEscale(Urban > \gamma_2)$	0.442***			
	(0.034)			
$FEscale(FEother \le \gamma_1)$		0.231***		
		(0.040)		
$FEscale(\gamma_1 < FEother \le \gamma_2)$		0.300***		
		(0.042)		
$FEscale(FEother > \gamma_2)$		0.421***		
		(0.041)		
$FEstructure\ (Urban \le \gamma_1)$			-0.005***	
			(0.001)	
$FEstructure\ (Urban > \gamma_1)$			-0.002	
			(0.001)	
Constant	0.093**	0.125***	0.154***	
	(0.009)	(0.009)	(0.009)	
F	100.54	86.86	73.89	
	(0.0000)	(0.0000)	(0.0000)	
R2	0.6548	0.6210	0.5437	

Note: *** p<0.01, ** p<0.05, * p<0.1. Standard errors in parentheses.

PTR Model 13- PTR Model 24.

S18 Table. The tests of threshold effect of the monocentric structure for PTR Model 13-Model 24, corresponding to the Fig. 4 of the manuscript.

Explained Variable	Explaining Variable	Threshold Variable	The type of threshold effect	F	P	Critical Value		
						10%	5%	1%
AE	*FEscale*	*Urban*	*Single*	91.62	0.000	26.541	31.449	46.052
			Double	36.94	0.008	20.454	25.959	33.653
			Triple	12.55	0.638	30.824	35.047	49.410
		FEother	*Single*	87.28	0.000	18.896	22.074	30.932
			Double	33.87	0.003	13.065	15.576	22.584
			Triple	15.24	0.310	22.738	26.709	34.194
	FEstructure	*Urban*	*Single*	49.59	0.023	24.080	34.971	69.025
			Double	17.52	0.183	24.895	32.183	42.749
			Triple	16.94	0.313	32.009	41.734	65.062
		FEother	*Single*	30.82	0.010	15.193	18.206	29.005
			Double	12.96	0.110	13.282	16.623	24.805
			Triple	4.95	0.660	12.723	15.844	20.391
IAE	*FEscale*	*Urban*	*Single*	96.87	0.000	16.644	21.993	41.382
			Double	18.58	0.125	20.393	54.797	102.343
			Triple	4.67	0.745	18.990	22.917	35.085
		FEother	*Single*	30.86	0.050	23.166	30.031	45.635
			Double	11.46	0.305	21.241	31.666	44.825
			Triple	10.28	0.263	19.525	27.813	49.771
	FEstructure	*Urban*	*Single*	81.01	0.000	17.897	22.433	44.687
			Double	21.50	0.130	27.641	45.098	87.501
			Triple	10.73	0.160	12.903	16.714	26.200
		FEother	*Single*	17.50	0.183	23.508	27.325	43.231
			Double	6.77	0.595	19.617	23.032	33.991
			Triple	5.01	0.675	14.411	19.965	30.888
PAE	*FEscale*	*Urban*	*Single*	67.98	0.005	35.659	41.533	57.004
			Double	12.95	0.525	31.327	41.399	59.277
			Triple	13.35	0.648	34.550	40.642	59.332
		FEother	*Single*	51.12	0.003	23.223	28.212	40.934
			Double	25.35	0.015	17.752	20.489	27.914
			Triple	8.05	0.630	19.203	23.918	32.688

	FEstructure	*Urban*	*Single*	35.29	0.093	34.252	46.397	73.280
			Double	7.92	0.668	27.726	34.627	54.702
			Triple	8.48	0.593	24.076	29.582	47.242
		FEother	*Single*	17.50	0.210	23.051	30.841	49.950
			Double	6.77	0.545	18.239	22.012	34.722
			Triple	5.01	0.698	14.394	20.144	33.349

S19 Table. The results of threshold effect of the monocentric structure for PTR Model 13-Model 24.

Variable	M13	M14	M15	M16
$\boldsymbol{LGS}$	-0.004	-0.269***	0.040	-0.080
	(0.064)	(0.066)	(0.077)	(0.079)
$\boldsymbol{IS}$	-0.023**	-0.023**	-0.048***	-0.052***
	(0.009)	(0.009)	(0.010)	(0.010)
$\boldsymbol{Open}$	0.001	0.024	0.011	-0.013
	(0.016)	(0.016)	(0.020)	(0.019)
$\boldsymbol{PI}$	-0.002	0.001	2.55e-05	-0.001
	(0.002)	(0.002)	(0.002)	(0.002)
$\boldsymbol{FEscale(Urban \leq \gamma_1)}$	-0.047			
	(0.083)			
$\boldsymbol{FEscale(\gamma_1 < Urban \leq \gamma_2)}$	0.160**			
	(0.077)			
$\boldsymbol{FEscale(Urban > \gamma_2)}$	0.386***			
	(0.076)			
$\boldsymbol{FEscale(FEother \leq \gamma_1)}$		-0.048		
		(0.075)		
$\boldsymbol{FEscale(\gamma_1 < FEother \leq \gamma_2)}$		0.083		
		(0.072)		
$\boldsymbol{FEscale(FEother > \gamma_2)}$		0.423***		
		(0.078)		
$\boldsymbol{FEstructure\ (Urban \leq \gamma_1)}$			-0.011***	
			(0.003)	
$\boldsymbol{FEstructure\ (Urban > \gamma_1)}$			0.002	
			(0.002)	
$\boldsymbol{FEstructure(FEother \leq \gamma_1)}$				-0.005**
				(0.003)
$\boldsymbol{FEstructure\ (FEother > \gamma_1)}$				0.002
				(0.002)
Constant	0.280***	0.303***	0.333***	0.357***

	(0.018)	(0.018)	(0.019)	(0.018)
F	28.05	28.47	13.11	13.35
	(0.0000)	(0.0000)	(0.0000)	(0.0000)
R2	0.4449	0.4485	0.2422	0.2456

Note: *** p<0.01, ** p<0.05, * p<0.1. Standard errors in parentheses.

S20 Table. The results of threshold effect of the monocentric structure for PTR Model 5-Model 8.

Variable	M17	M18	M19	M20
LGS	0.269***	0.068	0.370***	No threshold value
	(0.083)	(0.093)	(0.087)	
IS	0.005	-0.013	-0.009	
	(0.012)	(0.013)	(0.012)	
Open	0.028	0.017	0.069***	
	(0.020)	(0.022)	(0.022)	
PI	0.002	-0.001	0.001	
	(0.002)	(0.002)	(0.002)	
$\boldsymbol{FEscale(Urban \le \gamma_1)}$	-0.355***			
	(0.106)			
$\boldsymbol{FEscale(Urban > \gamma_1)}$	0.127			
	(0.091)			
$\boldsymbol{FEscale(FEother \le \gamma_1)}$		0.112		
		(0.101)		
$\boldsymbol{FEscale(FEother > \gamma_1)}$		0.292***		
		(0.103)		
$\boldsymbol{FEstructure\ (Urban \le \gamma_1)}$			-0.024***	
			(0.004)	
$\boldsymbol{FEstructure\ (Urban > \gamma_1)}$			0.001	
			(0.003)	
Constant	0.285***	0.312***	0.308***	
	(0.023)	(0.025)	(0.021)	
F	19.15	7.87	16.11	
	(0.0000)	(0.0000)	(0.0000)	
R2	0.3184	0.1610	0.2820	

Note: *** p<0.01, ** p<0.05, * p<0.1. Standard errors in parentheses.

S21 Table. The results of threshold effect of the monocentric structure for PTR Model 9-Model 12.

Variable	M21	M22	M23	M24
LGS	-0.064	-0.198***	0.117**	No threshold value
	(0.040)	(0.041)	(0.047)	
IS	-0.008	-0.008	-0.017***	
	(0.006)	(0.007)	(0.006)	
Open	-0.046***	-0.024**	-0.015	
	(0.010)	(0.010)	(0.011)	
PI	0.011***	0.012***	0.013***	
	(0.001)	(0.001)	(0.001)	
$\boldsymbol{FEscale(Urban \le \gamma_1)}$	0.272***			
	(0.044)			
$\boldsymbol{FEscale(Urban > \gamma_1)}$	0.419***			
	(0.047)			
$\boldsymbol{FEscale(FEother \le \gamma_1)}$		0.214***		
		(0.045)		
$\boldsymbol{FEscale(\gamma_1 < FEother \le \gamma_2)}$		0.284***		
		(0.045)		
$\boldsymbol{FEscale(FEother > \gamma_2)}$		0.423***		
		(0.046)		
$\boldsymbol{FEstructure\ (Urban \le \gamma_1)}$			-0.012***	
			(0.002)	
$\boldsymbol{FEstructure\ (Urban > \gamma_1)}$			-0.004***	
			(0.001)	
Constant	0.094***	0.109***	0.154***	
	(0.012)	(0.011)	(0.011)	
F	84.52	75.51	63.69	
	(0.0000)	(0.0000)	(0.0000)	
R2	0.6734	0.6833	0.6084	

Note: *** p<0.01, ** p<0.05, * p<0.1. Standard errors in parentheses.

PTR Model 25- PTR Model 36.

S22 Table. The tests of threshold effect of the polycentric structure for PTR Model 25-Model 36, corresponding to the Fig. 5 of the manuscript.

Explained Variable	Explaining Variable	Threshold Variable	The type of threshold effect	F	P	Critical Value		
						10%	5%	1%
AE	*FEscale*	*Urban*	*Single*	13.98	0.180	17.964	22.335	22.335
			Double	1.63	0.938	21.950	28.221	47.579
			Triple	1.76	0.878	14.036	21.595	33.295
		FEother	*Single*	29.39	0.028	20.196	24.865	35.100
			Double	11.01	0.318	20.175	24.358	39.361
			Triple	13.36	0.558	32.742	39.874	54.871
	FEstructure	*Urban*	*Single*	11.80	0.385	21.611	26.116	39.734
			Double	4.86	0.570	22.329	28.118	40.025
			Triple	2.16	0.855	13.372	18.341	33.438
		FEother	*Single*	26.99	0.050	19.804	26.943	32.725
			Double	9.40	0.358	16.732	21.779	28.606
			Triple	7.37	0.433	14.725	18.204	26.703
IAE	*FEscale*	*Urban*	*Single*	11.25	0.035	8.587	10.328	14.363
			Double	11.79	0.045	8.639	11.330	17.391
			Triple	8.88	0.573	25.672	30.888	41.647
		FEother	*Single*	14.75	0.093	14.326	16.664	21.961
			Double	4.54	0.383	8.286	9.810	14.789
			Triple	2.61	0.658	9.654	12.367	21.203
	FEstructure	*Urban*	*Single*	15.92	0.003	7.627	9.172	12.587
			Double	5.02	0.285	8.436	11.042	19.113
			Triple	9.66	0.370	20.090	26.416	36.839
		FEother	*Single*	4.24	0.500	8.709	10.324	12.895
			Double	2.20	0.768	7.418	9.801	13.692
			Triple	2.19	0.800	9.141	12.084	27.050
PAE	*FEscale*	*Urban*	*Single*	47.42	0.010	25.633	30.798	46.471
			Double	18.25	0.150	20.797	25.245	53.804
			Triple	17.70	0.320	44.712	55.152	81.341
		FEother	*Single*	22.29	0.008	12.350	15.417	20.319
			Double	4.83	0.640	11.831	15.054	24.049
			Triple	2.09	0.920	9.359	10.968	17.936

	FEstructure	*Urban*	*Single*	50.79	0.020	32.430	39.818	56.746
			Double	20.10	0.140	24.175	29.551	47.563
			Triple	16.02	0.295	28.770	38.262	48.489
		FEother	*Single*	28.34	0.000	13.941	18.143	24.276
			Double	2.76	0.923	10.853	12.963	18.807
			Triple	2.21	0.915	9.778	11.376	17.850

S23 Table. The results of threshold effect of the polycentric structure for PTR Model 25-Model 28.

Variable	M25	M26	M27	M28
LGS	No threshold value	-0.567***	No threshold value	-0.619***
		(0.074)		(0.076)
IS		-0.052***		-0.066***
		(0.007)		(0.008)
Open		-0.013		-0.015
		(0.015)		(0.015)
PI		0.001		0.001
		(0.001)		(0.001)
$\boldsymbol{FEscale(FEother \leq \gamma_1)}$		0.251**		
		(0.101)		
$\boldsymbol{FEscale(FEother > \gamma_1)}$		0.084		
		(0.109)		
$\boldsymbol{FEstructure(FEother \leq \gamma_1)}$				0.009***
				(0.003)
$\boldsymbol{FEstructure\ (FEother > \gamma_1)}$				0.002
				(0.002)
Constant		0.263***		0.290***
		(0.022)		(0.016)
F		37.64		33.80
		(0.0000)		(0.0000)
R2		0.6530		0.6283

Note: *** p<0.01, ** p<0.05, * p<0.1. Standard errors in parentheses.

S24 Table. The results of threshold effect of the polycentric structure for PTR Model 29-Model 32.

Variable	M29	M30	M31	M32
LGS	0.477***	0.043	0.569***	No threshold
	(0.174)	(0.182)	(0.172)	

IS	0.030*	0.009	0.028	value
	(0.018)	(0.016)	(0.018)	
Open	-0.034	-0.028	-0.035	
	(0.035)	(0.033)	(0.034)	
PI	0.002	-0.001	0.001	
	(0.002)	(0.002)	(0.002)	
$\boldsymbol{FEscale(Urban \leq \gamma_1)}$	0.404			
	(0.283)			
$\boldsymbol{FEscale(\gamma_1 < Urban \leq \gamma_2)}$	0.376			
	(0.255)			
$\boldsymbol{FEscale(Urban > \gamma_2)}$	0.480*			
	(0.245)			
$\boldsymbol{FEscale(FEother \leq \gamma_1)}$		0.716***		
		(0.234)		
$\boldsymbol{FEscale(FEother > \gamma_1)}$		0.973***		
		(0.256)		
$\boldsymbol{FEstructure\ (Urban \leq \gamma_1)}$			-0.007	
			(0.005)	
$\boldsymbol{FEstructure\ (Urban > \gamma_1)}$			-0.002	
			(0.00538)	
Constant	0.085*	0.099**	0.166***	
	(0.051)	(0.049)	(0.035)	
F	2.97	5.40	3.49	
	(0.0000)	(0.0000)	(0.0032)	
R2	0.1488	0.2125	0.1485	

Note: *** p<0.01, ** p<0.05, * p<0.1. Standard errors in parentheses.

S25 Table. The results of threshold effect of the polycentric structure for PTR Model 33-Model 36.

Variable	M33	M34	M35	M36
LGS	-0.044	-0.062	0.008	-0.016
	(0.045)	(0.050)	(0.046)	(0.050)
IS	-0.008	-0.024***	-0.012**	-0.024***
	(0.005)	(0.005)	(0.005)	(0.005)
Open	0.045***	0.058***	0.046***	0.058***
	(0.009)	(0.010)	(0.009)	(0.010)
PI	0.007***	0.008***	0.006***	0.007***
	(0.001)	(0.001)	(0.001)	(0.001)

$\boldsymbol{FEscale(Urban \le \gamma_1)}$	0.161**			
	(0.063)			
$\boldsymbol{FEscale(Urban > \gamma_1)}$	0.264***			
	(0.063)			
$\boldsymbol{FEscale(FEother \le \gamma_1)}$		0.211***		
		(0.069)		
$\boldsymbol{FEscale(FEother > \gamma_1)}$		0.131*		
		(0.072)		
$\boldsymbol{FEstructure\ (Urban \le \gamma_1)}$			-0.004**	
			(0.001)	
$\boldsymbol{FEstructure\ (Urban > \gamma_1)}$			-9.88e-05	
			(0.001)	
$\boldsymbol{FEstructure(FEother \le \gamma_1)}$				0.001
				(0.002)
$\boldsymbol{FEstructure\ (FEother > \gamma_1)}$				-0.003**
				(0.002)
Constant	0.056***	0.065***	0.097***	0.096***
	(0.014)	(0.015)	(0.009)	(0.011)
F	74.07	56.31	73.50	57.19
	(0.0000)	(0.0000)	(0.0000)	(0.0000)
R2	0.7874	0.7379	0.7861	0.7409

Note: *** p<0.01, ** p<0.05, * p<0.1. Standard errors in parentheses.

References:

[S1] Parui P. Fiscal expansion, government debt and economic growth: a post-Keynesian perspective. Journal of Post Keynesian Economics. 2024, 47: 117-154. https://doi.org/10.1080/01603477.2023.2221444.

[S2] Tosun MS, Abizadeh S. Economic growth and tax components: an analysis of tax changes in OECD. Applied Economics. 2005; 37: 2251-2263. https://doi.org/10.1080/00036840500293813.

[S3] Adkisson RV, Mohammed M. Tax structure and state economic growth during the Great Recession. Social Science Journal. 2014; 51: 79-89. https://doi.org/10.1016/j.soscij.2013.10.009.

[S4] Erum N, Sohag K, Said J, Musa K, Asghar MM. Governance, fiscal expenditure, and economic growth in OIC countries: Role of natural resources and information communication technology. Resources Policy. 2024; 90: 104717. https://doi.org/10.1016/j.resourpol.2024.104717.Adams REW, Jones RC. Spatial Patterns and Regional Growth among Classic Maya Cities. American Antiquity. 1981;46(2):301-322. https://doi.org/10.2307/280210.

[S5] Adams REW, Jones RC. Spatial Patterns and Regional Growth among Classic Maya Cities. American Antiquity. 1981;46(2):301-322. https://doi.org/10.2307/280210.

[S6] Hansen, B. E. (1999). "Threshold effects in non-dynamic panels: Estimation, testing, and inference," Journal of Econometrics, 93(2), 345-368. https://doi.org/10.1016/S0304-4076(99)00025-1.

[S7] Caner, M and Hansen, BE. 2004 “Instrumental variable estimation of a threshold model.” Econometric Theory (20): 813-843. https://doi.org/10.1017/S0266466604205011.
